# Supplementary material for: The spatiotemporal evolution of flight-coupled wind field for a four-rotor plant protection unmanned aerial vehicle
Source: PLoS One. 2025 May 8;20(5):e0318769. doi: 10.1371/journal.pone.0318769 (PMC12061431; doi:10.1371/journal.pone.0318769)
Supplement: Attachment — Submitted filename: PONE-D-24-27043_reviewer.pdf [file pone.0318769.s001.pdf]

PLOS ONE

The Spatiotemporal Evolution of Flight-Coupled Wind Field for a Four-Rotor Plant Protection Unmanned Aerial Vehicle

--Manuscript Draft--

**Manuscript Number:**	PONE-D-24-27043
**Article Type:**	Research Article
**Full Title:**	The Spatiotemporal Evolution of Flight-Coupled Wind Field for a Four-Rotor Plant Protection Unmanned Aerial Vehicle
**Short Title:**	The Spatiotemporal Evolution of Flight-Coupled Wind Field for a Four-Rotor Plant Protection UAV
**Corresponding Author:**	Fengbo Yang<br>Nanjing Forestry University<br>Nanjing, CHINA
**Keywords:**	unmanned aerial vehicle; flight-coupled wind field; spatiotemporal evolution; wind field test; computational fluid dynamics; dynamic mesh; interface.
**Abstract:**	The four-rotor plant protection Unmanned Aerial Vehicle (UAV) is an important piece of equipment for the efficient plant protection field. However, the flight-coupled wind is unclear, which has become the bottleneck factor limiting the improvement of the spray quality. Only when the coupled wind field is analyzed can the interaction mechanism among wind field, pesticide droplet, and crop canopy be effectively studied, then the flight spray scheme can be reasonably formulated. By combining Re-Normalization Group (RNG) k-ε turbulence model, compressible Reynolds Averaged Navier-Stokes (RANS) equations, dynamic mesh based on the spring smoothing and layering method, pressure-velocity coupling algorithm, this paper established a computational fluid dynamics (CFD) model of the flight-coupled wind field for the four-rotor plant protection UAV and discussed the dynamic evolution law of the flight-coupled wind field in the spatial and temporal dimensions. Numerical simulations were carried out for flight-coupled wind fields in two working conditions, analysis showed that the relative error between the simulated and measured values of Zb-direction velocity was less than 12.8% when the flight-coupled wind field was stable. When switching from hover to flight state, the downwash wind field experienced a lateral interruption and developed into the stable flight-coupled wind field after flying for 0.696 s, and the velocity distribution diagram of the cross section evolved from four rings to four horseshoe vortices. When the flight-coupled wind field evolved to stabilize, the dense atmosphere reduced the absolute values of the four Zb-direction velocity peaks at observation line 1 from left to right along the flight direction by 4.5%, 4.2%, 9.0%, and 26.1% respectively. The angles between the horizontal direction and the four curves formed by Zb-direction velocity peaks were also changed from 90° to 72°, 69°, 61°, and 56° respectively at the flight speed of 3m/s. Therefore, the installation of the nozzle and the formulation of the spraying strategy are crucial to improve the spray deposition effect.
**Order of Authors:**	Fengbo Yang
	Zhiwei Li
	Guangyao Zhang
	Hongping Zhou
**Additional Information:**	
**Question**	**Response**
**Financial Disclosure**<br><br>Enter a financial disclosure statement that describes the sources of funding for the work included in this submission. Review	Yes

Powered by Editorial Manager® and ProduXion Manager® from Aries Systems Corporation

the submission guidelines for detailed requirements. View published research articles from *PLOS ONE* for specific examples.<br><br>This statement is required for submission and **will appear in the published article** if the submission is accepted. Please make sure it is accurate.<br><br>**Funded studies**<br>Enter a statement with the following details:<br>• Initials of the authors who received each award<br>• Grant numbers awarded to each author<br>• The full name of each funder<br>• URL of each funder website<br>• Did the sponsors or funders play any role in the study design, data collection and analysis, decision to publish, or preparation of the manuscript?<br><br>Did you receive funding for this work?	
Please add funding details.<br>as follow-up to "**Financial Disclosure**<br><br>Enter a financial disclosure statement that describes the sources of funding for the work included in this submission. Review the submission guidelines for detailed requirements. View published research articles from *PLOS ONE* for specific examples.<br><br>This statement is required for submission and **will appear in the published article** if the submission is accepted. Please make sure it is accurate.	The authors acknowledge the support provided by the National Natural Science Foundation of China (No. 52275257, No. 51705264)

**Funded studies**<br>Enter a statement with the following details:<br>• Initials of the authors who received each award<br>• Grant numbers awarded to each author<br>• The full name of each funder<br>• URL of each funder website<br>• Did the sponsors or funders play any role in the study design, data collection and analysis, decision to publish, or preparation of the manuscript?<br><br>Did you receive funding for this work?"	
Please select the country of your main research funder (please select carefully as in some cases this is used in fee calculation).<br>as follow-up to "**Financial Disclosure**<br><br>Enter a financial disclosure statement that describes the sources of funding for the work included in this submission. Review the submission guidelines for detailed requirements. View published research articles from *PLOS ONE* for specific examples.<br><br>This statement is required for submission and **will appear in the published article** if the submission is accepted. Please make sure it is accurate.<br><br>**Funded studies**<br>Enter a statement with the following details:<br>• Initials of the authors who received each award<br>• Grant numbers awarded to each author<br>• The full name of each funder<br>• URL of each funder website<br>• Did the sponsors or funders play any role in the study design, data collection and analysis, decision to publish, or preparation of the manuscript?<br><br>Did you receive funding for this work?"	CHINA - CN
**Competing Interests**	The authors declare no conflict of interest

Use the instructions below to enter a competing interest statement for this submission. On behalf of all authors, disclose any competing interests that could be perceived to bias this work—acknowledging all financial support and any other relevant financial or non-financial competing interests.<br><br>This statement is required for submission and **will appear in the published article** if the submission is accepted. Please make sure it is accurate and that any funding sources listed in your Funding Information later in the submission form are also declared in your Financial Disclosure statement.<br><br>View published research articles from *PLOS ONE* for specific examples.<br><br>**NO authors have competing interests**<br><br>Enter: *The authors have declared that no competing interests exist.*<br><br>**Authors with competing interests**<br><br>Enter competing interest details beginning with this statement:<br><br>*I have read the journal's policy and the authors of this manuscript have the following competing interests: [insert competing interests here]*<br><br>* typeset	
**Ethics Statement**<br><br>Enter an ethics statement for this submission. This statement is required if the study involved:<br><br>• Human participants<br>• Human specimens or tissue<br>• Vertebrate animals or cephalopods<br>• Vertebrate embryos or tissues	N/A

Powered by Editorial Manager® and ProduXion Manager® from Aries Systems Corporation

• Field research<br><br>Write "N/A" if the submission does not require an ethics statement.<br><br>General guidance is provided below. Consult the submission guidelines for detailed instructions. **Make sure that all information entered here is included in the Methods section of the manuscript.**	

Powered by Editorial Manager® and ProduXion Manager® from Aries Systems Corporation

**Format for specific study types**<br><br>**Human Subject Research (involving human participants and/or tissue)**<br>• Give the name of the institutional review board or ethics committee that approved the study<br>• Include the approval number and/or a statement indicating approval of this research<br>• Indicate the form of consent obtained (written/oral) or the reason that consent was not obtained (e.g. the data were analyzed anonymously)<br><br>**Animal Research (involving vertebrate animals, embryos or tissues)**<br>• Provide the name of the Institutional Animal Care and Use Committee (IACUC) or other relevant ethics board that reviewed the study protocol, and indicate whether they approved this research or granted a formal waiver of ethical approval<br>• Include an approval number if one was obtained<br>• If the study involved *non-human primates*, add *additional details* about animal welfare and steps taken to ameliorate suffering<br>• If anesthesia, euthanasia, or any kind of animal sacrifice is part of the study, include briefly which substances and/or methods were applied<br><br>**Field Research**<br><br>Include the following details if this study involves the collection of plant, animal, or other materials from a natural setting:<br>• Field permit number<br>• Name of the institution or relevant body that granted permission	
**Data Availability**<br><br>Authors are required to make all data underlying the findings described fully available, without restriction, and from the time of publication. PLOS allows rare exceptions to address legal and ethical concerns. See the PLOS Data Policy and FAQ for detailed information.	No - some restrictions will apply

A Data Availability Statement describing where the data can be found is required at submission. Your answers to this question constitute the Data Availability Statement and **will be published in the article**, if accepted.<br><br>**Important:** Stating 'data available on request from the author' is not sufficient. If your data are only available upon request, select 'No' for the first question and explain your exceptional situation in the text box.<br><br>Do the authors confirm that all data underlying the findings described in their manuscript are fully available without restriction?	
**Describe where the data may be found in full sentences. If you are copying our sample text, replace any instances of XXX with the appropriate details.**<br><br>• If the data are **held or will be held in a public repository**, include URLs, accession numbers or DOIs. If this information will only be available after acceptance, indicate this by ticking the box below. For example: *All XXX files are available from the XXX database (accession number(s) XXX, XXX.)*.<br>• If the data are all contained **within the manuscript and/or Supporting Information files**, enter the following: *All relevant data are within the manuscript and its Supporting Information files.*<br>• If neither of these applies but you are able to provide **details of access elsewhere**, with or without limitations, please do so. For example:<br><br>*Data cannot be shared publicly because of [XXX]. Data are available from the XXX Institutional Data Access / Ethics Committee (contact via XXX) for researchers who meet the criteria for access to confidential data.*<br><br>*The data underlying the results presented in the study are available from (include the name of the third party*	All relevant data are within the manuscript and its Supporting Information files.

Powered by Editorial Manager® and ProduXion Manager® from Aries Systems Corporation

and contact information or URL).<br>• This text is appropriate if the data are owned by a third party and authors do not have permission to share the data.<br><br>* typeset	
Additional data availability information:	

Manuscript

Click here to access/download;Manuscript;PLOS ONE-7-02-ppp-ok.doc

The Spatiotemporal Evolution of Flight-Coupled Wind Field for a Four-Rotor Plant Protection Unmanned Aerial Vehicle

Yang Fengbo*, Li Zhiwei, Zhang Guangyao, Zhou Hongping

College of Mechanical and Electronic Engineering, Nanjing Forestry University, Nanjing 210037, China
*Correspondence: yfb@njfu.edu.cn

Abstract: The four-rotor plant protection Unmanned Aerial Vehicle (UAV) is an important piece of equipment for the efficient plant protection field. However, the flight-coupled wind is unclear, which has become the bottleneck factor limiting the improvement of the spray quality. Only when the coupled wind field is analyzed can the interaction mechanism among wind field, pesticide droplet, and crop canopy be effectively studied, then the flight spray scheme can be reasonably formulated. By combining Re-Normalization Group (RNG) k-ε turbulence model, compressible Reynolds Averaged Navier-Stokes (RANS) equations, dynamic mesh based on the spring smoothing and layering method, pressure-velocity coupling algorithm, this paper established a computational fluid dynamics (CFD) model of the flight-coupled wind field for the four-rotor plant protection UAV and discussed the dynamic evolution law of the flight-coupled wind field in the spatial and temporal dimensions. Numerical simulations were carried out for flight-coupled wind fields in two working conditions, analysis showed that the relative error between the simulated and measured values of Z_b-direction velocity was less than 12.8% when the flight-coupled wind field was stable. When switching from hover to flight state, the downwash wind field experienced a lateral interruption and developed into the stable flight-coupled wind field after flying for 0.696 s, and the velocity distribution diagram of the cross section evolved from four rings to four horseshoe vortices. When the flight-coupled wind field evolved to stabilize, the dense atmosphere reduced the absolute values of the four Z_b-direction velocity peaks at observation line 1 from left to right along the flight direction by 4.5%, 4.2%, 9.0%, and 26.1% respectively. The angles between the horizontal direction and the four curves formed by Z_b-direction velocity peaks were also changed from 90° to 72°, 69°, 61°, and 56° respectively at the flight speed of 3m/s. Therefore, the installation of the nozzle and the formulation of the spraying strategy are crucial to improve the spray deposition effect.

1. Introduction

Multi-rotor plant protection Unmanned Aerial Vehicle (UAV), which has broken through the constraint factors of planting terrains and crop types, has the ability that traditional ground self-propelled and manual plant protection equipment cannot complete [1-4]. Therefore, due to the vast territory and diverse planting terrain, multi-rotor UAVs have been widely used in China, especially four-rotor and hexa-rotor UAVs [5-6]. By the end of 2021, the number of plant protection UAVs is about 120000 in China, and the annual spray area is about 1070000000 mu. Up to now, multi-rotor UAVs have become one of the indispensable plant protection equipment in China [7-8].

The research results show that the flight-coupled wind field composed of upwind airflow, downwash of the rotors, and natural crosswinds will significantly affect the spray deposition effect of plant protection UAVs. The horizontal coverage width, vertical wind speed magnitude, and spatiotemporal evolution of the flight-coupled wind field are the critical factors that affect the deposition level and spray coverage of the target [5, 9-10]. Deposition uniformity and drift under the flight-coupled wind field are typical problems faced by aviation spray (as shown in Fig 1). Under the downforce of the flight-coupled airflow, the droplet swarm is transported to the crop canopy area, and the crop canopy moves laterally. If the deposition of droplets on the canopy and the lateral movement of crops occurred simultaneously, the uniformity of deposition on both sides of leaves would be significantly improved [11]. So the flight-coupled wind field is a key factor affecting the deposition and drift levels of the multi-rotor plant protection UAV [12]. Therefore, related research has been conducted by scholars on the distribution characteristics of the flight-coupled airflow for multi-rotor plant protection UAVs, and the relationship between airflow distribution and droplet deposition. Of course, analyzing the dynamic distribution of the flight-coupled wind field during flight is the first crucial step.

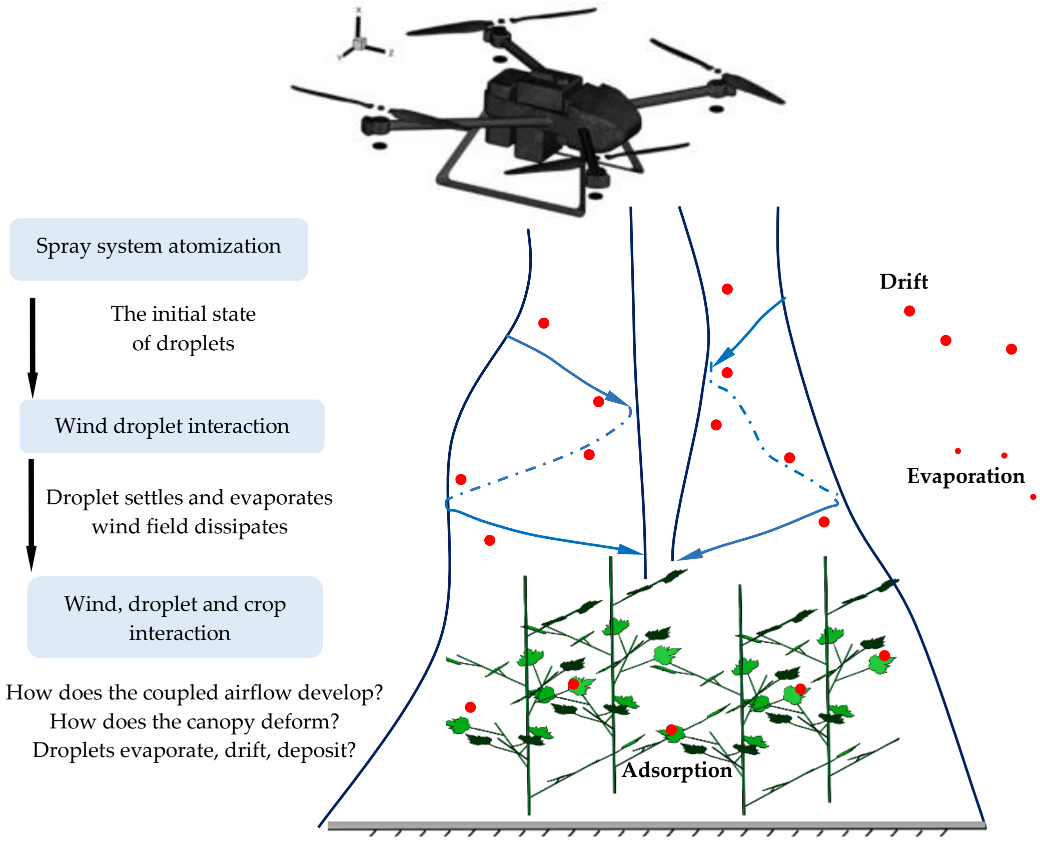

Fig 1. Schematic diagram of flight spray of four-rotor plant protection UAV.

Combining CFD numerical calculation with a wind speed test could well obtain the spatiotemporal characteristics of the flight-coupled wind field and analyze the causes of the flow law [4, 8]. The evolution law of wake vortices for the Thrush 510G over time, and the interaction between wake vortices and hard ground were simulated by Bin Zhang et al. [13] using the CFD method. Based on the gas-liquid multiphase flow model, the CFD technique was applied to analyze the wake vortices and the droplet motion trajectory for the Thrush 510G aircraft by Bin Zhang et al. [14]. Although the authors' research is only based on the two-dimensional model, we can observe the distribution details of the wake vortices and the motion trajectories of the droplet group in real time, which is impossible for the wind speed test. The aerodynamic interference characteristic between the tail-wing and the propeller-rotor, and its effect on the lateral stability were

researched for a tilt-rotor UAV by Jung et al. [15] during transient flight modes. The downwash of a hexa-rotor plant protection UAV in hover was calculated by Zhang et al. [16] in a virtual orchard, and the distribution laws of the downwash were analyzed in different natural crosswind speeds, fruit tree growth stages, and hover heights. The interaction mechanism between the flight-coupled airflow of a UAV and the vortex in the rice canopy was researched by Li et al. [17]. In the research results, to enhance the target precision of spray for the UAV, the theoretical model that included the UAV motion parameters and the vortex movement was also established [17].

In terms of parameter measurement of downwash airflow for plant protection UAVs, scholars have also conducted many effective studies. Setting the wireless micro-meteorology airflow speed sensors in a line, the flight-coupled airflow of a single-rotor UAV working in rice field was measured by Wang et al. [18]. According to the results of the tests, the conclusion could be drawn that the flight direction, height, and natural crosswind weakened the downwash intensity in the vertical direction. Wang et al. [19] measured the velocity of the flight-coupled airflow field in x, y, and z directions based on the application scenario of hybrid rice breeding supplementary pollination. The test experiment indicated that the relationship among the speed values was V_X (flight direction) > V_y (perpendicular to the xoz plane) > V_z (the vertical direction), and the airflow in the flight direction was the most useful to the supplementary pollination. Li et al. [20] set up three-direction airflow speed sensors in a line in the rice canopy, and then got the airflow velocity at several heights in the rice canopy. The results indicated that a "steep" effect existed in the velocity values in the UAV flight direction, which meant that the increasing rate of the speed value in the flight direction was higher than the reducing rate in the backward direction.

The flight-coupled airflow forces the droplet swarm to move, deposit, and drift; the flight-coupled airflow drives the crop canopy to bend; then the strong flight-coupled airflow develops into a faint wind. In conclusion, the flight-coupled airflow is a crucial factor for the multi-rotor UAV spray. However, research on the flight-coupled airflow of the plant protection UAV mainly focuses on the spatial scale, the detailed flow laws of flight-coupled airflow in the temporal scale are rarely mentioned. In addition, many studies on downwash and flight wind fields based on the CFD tools are limited to the rotor structure itself, the scientific research achievements of downwash and flight wind fields based on the overall plant protection UAV are also rarely mentioned. In plant protection, the CFD tool was used for the auxiliary analysis of orchard spray first [21-22]. However, the CFD tool has continued to be used in aviation spray in the past decade. Therefore, this paper took the overall structure of a four-rotor plant protection UAV as the study object, and studied the evolutionary law of flight wind field in the spatiotemporal dimensions by combining Reynolds Averaged Navier-Stokes equations, Re-Normalization Group k-ε turbulence model, dynamic mesh based on the spring smoothing and layering method, pressure-velocity coupling algorithm. The Z_b-direction wind speed test experiment was designed to measure the flow velocities of the flight-coupled wind field at the feature points for the four-rotor UAV, and the reliability of the CFD simulation was confirmed by fitting the simulated and measured values of Z_b-direction wind speed. The workflow of this paper is shown in Fig 2.

2. Methods and Models

2.1. Governing equations of the flight-coupled wind field

In this article, the governing equation in the flight-coupled airflow area was the Reynolds-Averaged Navier-Stokes for the four-rotor UAV. Due to the rotation of UAV rotors, a source term was added to the governing equation, which could be written as follows [23-24]:

$$\frac{\partial}{\partial t}\iiint_{\partial V}\vec{W}\,dV+\iint_{\partial S}\left(\vec{F}-\vec{G}\right)\cdot\vec{n}\,dS=\iiint_{\partial V}\vec{Q}\,dV \quad (1)$$

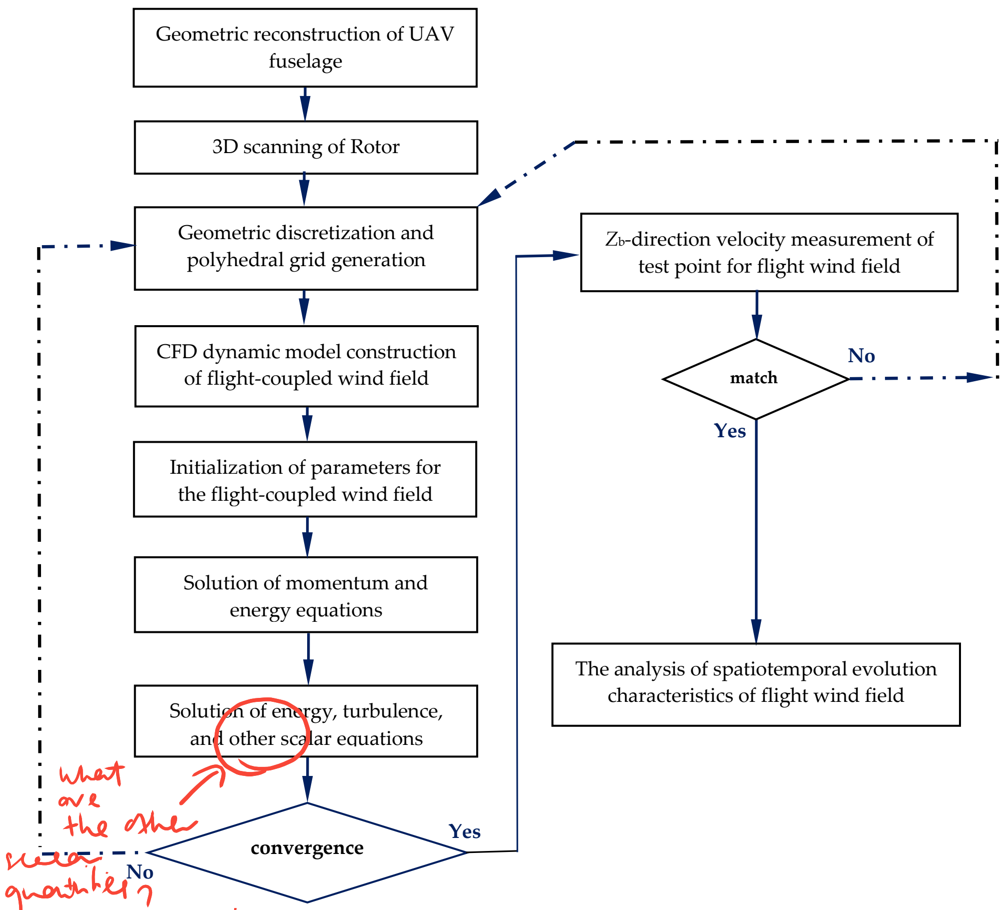

Fig 2. Roadmap of this paper.

$$\vec{W}=\begin{bmatrix}\rho\\ \rho u\\ \rho v\\ \rho\omega\\ \rho E_r\end{bmatrix},\quad \vec{F}=\begin{bmatrix}\rho(\vec{q}-\vec{q}_w)\\ \rho u(\vec{q}-\vec{q}_w)+p\vec{i}_x\\ \rho v(\vec{q}-\vec{q}_w)+p\vec{i}_y\\ \rho\omega(\vec{q}-\vec{q}_w)+p\vec{i}_z\\ \rho H(\vec{q}-\vec{q}_w)\end{bmatrix},\quad \vec{G}=\begin{bmatrix}0\\ \tau_{xx}\vec{i}_x+\tau_{yx}\vec{i}_y+\tau_{zx}\vec{i}_z\\ \tau_{xy}\vec{i}_x+\tau_{yy}\vec{i}_y+\tau_{zy}\vec{i}_z\\ \tau_{xz}\vec{i}_x+\tau_{yz}\vec{i}_y+\tau_{zz}\vec{i}_z\\ \Phi_x\vec{i}_x+\Phi_y\vec{i}_y+\Phi_z\vec{i}_z\end{bmatrix},\quad \vec{\boldsymbol{Q}}=\begin{bmatrix}0\\ -\rho\omega\Omega\\ 0\\ \rho u\Omega\\ 0\end{bmatrix} \tag{2}$$

Where V is the control body of the computational domain; S is the control surface of the control volume; $\overline{W}$ is the conservation vector of the equation; the $\overline{F}$ and $\overline{G}$ is the inviscid and viscous fluxes of the equation; $\overline{Q}$ is the source flux due to the source term; H and E_r are the total enthalpies and total internal energy of a mesh unit respectively; $\tau_{xx/yy/zz}$, $\tau_{xy/xz/yz}$, $\Phi_{x/y/z}$ are the viscous quantities in three directions respectively, $\overline{q}_w$ and $\overline{q}$ denote grid and air velocity perpendicular to the unit surface; u, v, and w are the air velocity components of a mesh unit in three directions respectively; ρ and p are the airflow density and pressure of a mesh unit.

2.2. Viscous model and numerical difference method

The Re-Normalization Group k-ε turbulence model [25] was adopted to calculate turbulent effects caused by large shear flow, and the transport equations of turbulent kinetic energy k equation and the ε equation were shown in equations 3 and 4.

k equation:

$$\frac{\partial(\rho k)}{\partial t}+\frac{\partial(\rho k u_i)}{\partial x_i}=\frac{\partial}{\partial x_j}\left[\alpha_k\mu_{eff}\frac{\partial k}{\partial x_j}\right]+G_{kb}-\rho\varepsilon \tag{3}$$

ε equation:

$$\frac{\partial(\rho\varepsilon)}{\partial t}+\frac{\partial(\rho\varepsilon u_i)}{\partial x_i}=\frac{\partial}{\partial x_j}\left[\alpha_k\mu_{eff}\frac{\partial\varepsilon}{\partial x_j}\right]+C^*_{1\varepsilon}\frac{\varepsilon}{k}G_k-G_{2\varepsilon}\rho\frac{\varepsilon^2}{k} \tag{4}$$

Where $\alpha_k=\alpha_\varepsilon=1.39$; $C_\mu=0.0845$; $C_{1\varepsilon}=1.42$; $C_{2\varepsilon}=1.68$; $\eta_0=4.377$; $\beta=0.012$; $\mu_{eff}=\mu+\mu_i$; $\mu_i=\rho C_\mu\frac{k^2}{\varepsilon}$; $C^*_{1\varepsilon}=C_{1\varepsilon}-\frac{\eta(1-\eta/\eta_0)}{1+\beta\eta^3}$; $\eta=\sqrt{2E_{ij}gE_{ij}}\frac{k}{\varepsilon}$; $E_{ij}=\frac{1}{2}\left(\frac{\partial u_i}{\partial x_j}+\frac{\partial u_j}{\partial x_i}\right)$.

The finite volume method was adopted to discretize the Reynolds-Averaged Navier-Stokes equations, then the second-order coupling scheme based on a pressure-based solver was used for calculation [25].

2.3. Structure, boundary condition, and mesh model

In this research, the flight-coupled wind field testing experiment has been strongly supported by XAIRCRAFT (Guangzhou, China). The 3D model construction process of the four-rotor UAV is shown in the following Fig 3. A Solidworks drawing tool was used to draw the main structure of the UAV (as shown in Fig 3b). The point cloud of the execution component UAV rotor was obtained by a 3D Optimscan 5-2015011 K05 scanner (as shown in Fig 3c). The Solidworks tool was used to synthesize the cloud point, and the complex surface of the UAV rotor was obtained (as shown in Fig 3e). Since the rotors are closer to the main structure, the flight-coupled wind field would be significantly affected by the UAV body structure, and the body structure and rotors should be assembled for the transient calculation of the flight-coupled wind field (as shown in Fig 3f).

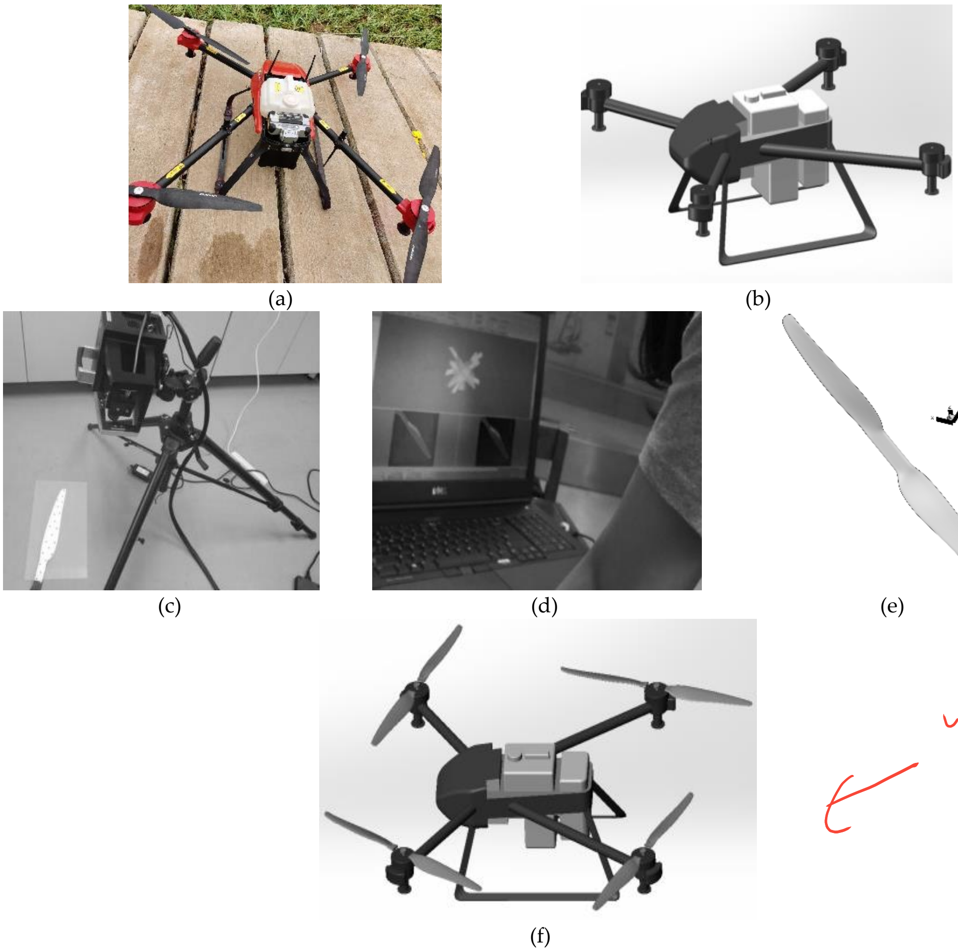

Fig 3. Scanning and reconstruction of the four-rotor UAV. (a) The actual structure of the four-rotor UAV; (b) Geometric reconstruction of the four-rotor UAV fuselage; (c) Calibrating and scanning the rotor; (d) Point cloud synthesis site; (e) View of the structured model of the rotor component; (f) The final 3D model of the four-rotor UAV.

The motion system diagram of the four-rotor plant protection UAV is shown in the following Fig 4. It can be seen from Fig 4 that to maintain the torque balance of the UAV body, the rotational speed between adjacent rotors is opposite. Rotor 1 and rotor 3 are set to rotate clockwise, while rotor 2 and rotor 4 are set to rotate counterclockwise. When the rotational speeds of the four rotors are equal, and the resultant force generated by the rotation of the four rotors is exactly equal to the weight of the UAV body, the UAV will be in hover state. Adjusting the rotation speed of the four rotors can realize the adjustment of flight speed and flight attitude. There were two coordinate systems in Fig 4, namely the absolute coordinate system $O_bX_bY_bZ_b$ and the relative coordinate system $O_eX_eY_eZ_e$; and the coordinate system was set according to the right-hand rule. The geometric center of each rotor was the coordinate origin of the respective relative coordinate system, and the three coordinate axes were parallel to the coordinate axes of the same name in the absolute coordinate system. The absolute coordinate system was used to set the position and rotation angular speed of each rotor, and the relative coordinate system was used to set the rotation axis of each rotor. When the four-rotor plant protection UAV flew along the negative direction of the X_b-axis in the absolute coordinate system, the speed of flight was 3m/s. When flying, the overall forward inclination of the UAV body was 9°, so the rotation axis of each rotor was not the Z_e-axis of the relative coordinate system. The rotation axis of each rotor was in the $O_eX_eZ_e$ plane of the relative coordinate system, and the coordinate values of the X_e-axis and Z_e-axis were -0.156434 and 0.987688.

In this paper, to better match the wind field test and find the evolution law of the dynamic flight flow field, the dynamic theoretical calculations of the flight wind field under two operating conditions were carried out. The first condition was that the UAV hovered for 1.3 s, and then flew in the X_b-axis negative direction at a speed of 3 m/s. The second condition was that the UAV flew directly along the X_b-axis negative direction at a speed of 3 m/s. In addition, in the two calculation conditions, the working parameters of each rotor are shown in Table 1 below. It was worth noting that due to the dynamic calculation, the working parameters of each rotor would be significantly different when the four-rotor plant protection UAV was fixed and flying. When the time was 0s, the rotation center and rotational angular velocity of the same rotor were the same in calculation conditions 1 and 2. In calculation condition 1, when the UAV body was fixed, the rotation center of each rotor remained unchanged; when flying at a speed of 3 m/s along the negative direction of the X_b-axis, the rotation center X_b-coordinate of each rotor continuously decreased, and the relationship between the X_b-coordinate and the calculation time t was shown in Table 1. In the calculation condition 2, the X_b-coordinate of the rotation center of each rotor kept decreasing.

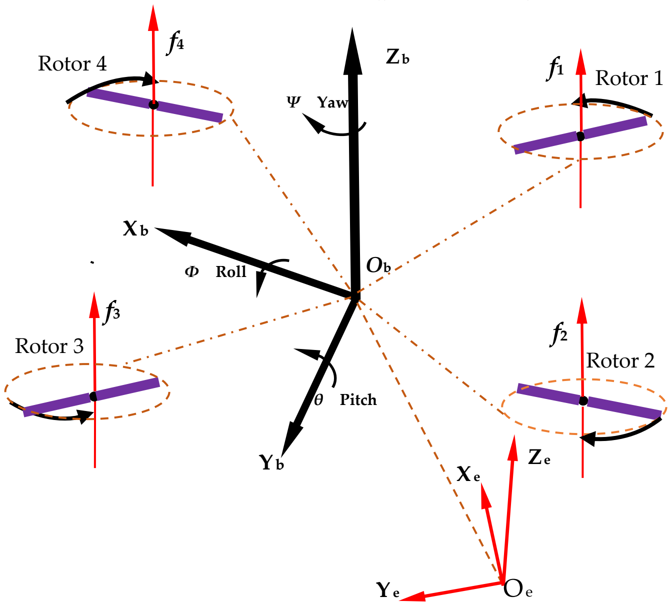

Fig 4. Four-rotor UAV motion system diagram.

Table 1. Working parameters of each rotor

Rotor number	Rotation center of calculation condition 1 (*x*/*m*; *y*/*m*; *z*/*m*.)	Rotation center of calculation condition 2 (*x*/*m*; *y*/*m*; *z*/*m*.)	Angular velocity of rotation (rad/s)
1	-0.415239839(fixed), -0.415239839-(3**t*/1[s]- 3*1.3)(flight); -0.518860346; -0.099685111.	-0.415239839-3**t*/1[s]; -0.518860346; -0.099685111.	249.9
2	-0.415239839(fixed), -0.415239839-(3**t*/1[s]- 3*1.3)(flight); 0.518860347; -0.099685111.	-0.415239839-3**t*/1[s]; 0.518860347; -0.099685111.	-249.9
3	0.541537512(fixed), 0.541537512-(3**t*/1[s]- 3*1.3)(flight); 0.516302113; 0.051853534.	0.541537512-3**t*/1[s]; 0.516302113; 0.051853534.	253
4	0.541537512(fixed), 0.541537512-(3**t*/1[s]- 3*1.3)(flight); -0.516302112; 0.051853534.	0.541537512-3**t*/1[s]; -0.516302112; 0.051853534.	-253

Fig 5 shows the boundary setting of the theoretical calculation model of the dynamic coupled wind field for the four-rotor plant protection unmanned UAV in this paper. The rotor surface, the UAV body, and the ground were all set as the solid wall boundary without slip. As could be seen from Fig 5, the entire computing domain was divided into four computing domains; the first area was a region of four small cylinders, each enclosing a rotor; the second was the hexahedral region that wrapped the UAV body; the third was the hexahedron region above that moved with the UAV; the fourth was the stationary hexahedron region close to the ground. Boolean operation was performed on the cylinder and rotor in the first area, and the solid domain of the rotor was deleted. In the second region, a Boolean operation was performed on the hexahedron and the UAV body, and the solid domain of the UAV body was deleted; an area that exactly corresponds to the spatial location of the first computational domain was dug out in the second computational domain, then computing domain 1 and computing domain 2 were connected and flow field data were exchanged through interface1. An area that exactly corresponds to the spatial location of the second computational domain was dug out in the third computational domain, then computing domain 2 and computing domain 3 were connected and flow field data were exchanged through interface 2. The lower-end face in the third computational domain was completely aligned with the upper-end face in the fourth computational domain, then computing domain 3 and computing domain 4 were connected and flow field data were exchanged through interface 3. In dynamic calculation, to ensure that the lower-end face of the third computing domain and the upper-end face of the fourth computing domain were always aligned, the surface directly opposite the negative direction of the X_b-axis in the third computing domain was the grid vanishing surface, and the surface directly opposite the positive direction of the X_b-axis was the grid generating surface, and the rate of grid generation and disappearance was 3m/s. In addition, the upper, front, back, left and right sides of the third computing domain were pressure outlet boundaries; and the front, back, left and right sides of the fourth computing domain were all also pressure outlet boundaries.

In general, through the setting of the above boundaries, the numerical calculation of the dynamic flow field of the four-rotor plant protection UAV moving along the negative direction of the X_b-axis at a speed of 3m/s could be realized.

does the location of the computational domain @ Domain 1 - Domain 4 corresponds to these 4 interfaces?

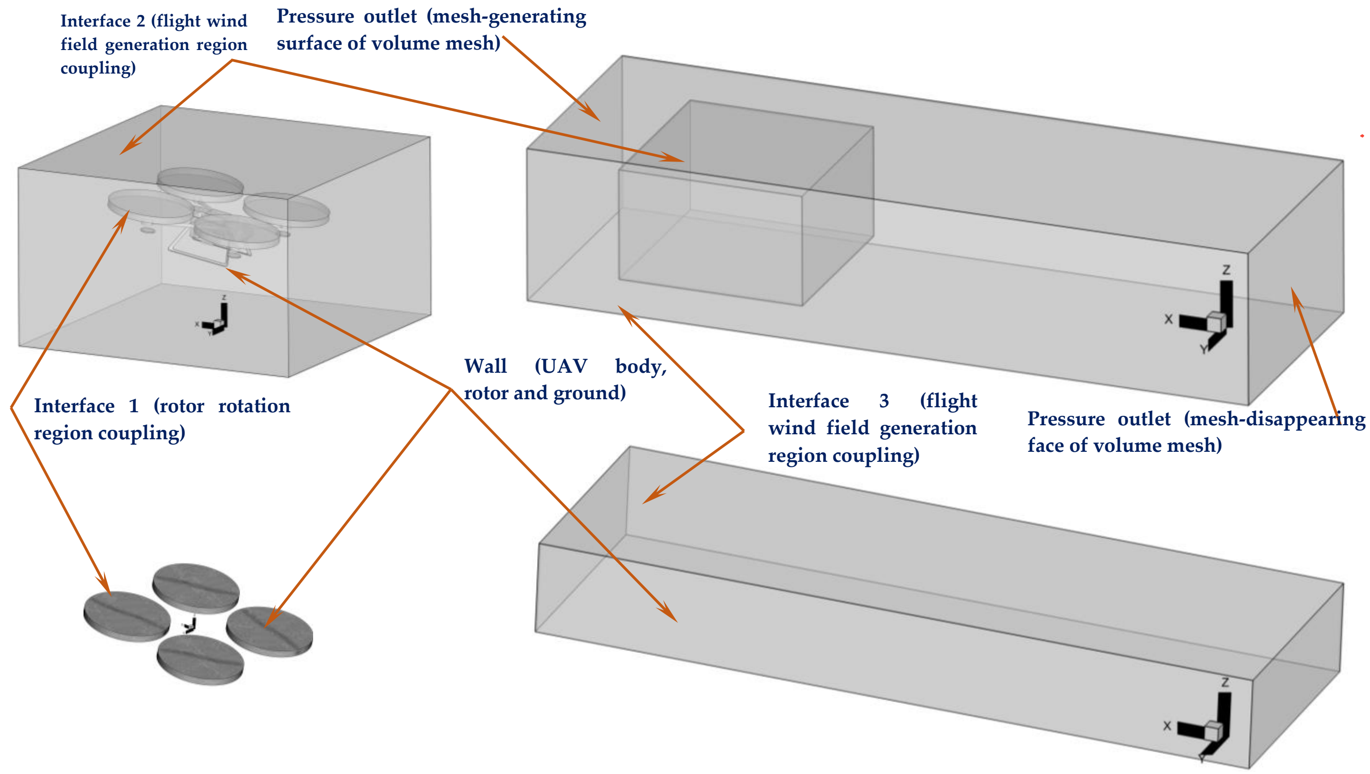

Fig 5. Schematic diagram of boundary condition setting.

To improve the calculation accuracy of the flight-coupled airflow, the grid number of the entire computing domain reached 4.598 million. The mesh models of computing domain 1 and computing domain 2 were constructed using the polyhedral mesh strategy, the mesh models of computing domain 3 and computing domain 4 were constructed using regular hexahedral structured mesh, and the minimum side length of the grid was 2 mm. The surface grid distribution law for the four-rotor UAV is shown in Fig 6. To show the rotor surface grid more clearly, the grid distribution near the area was enlarged.

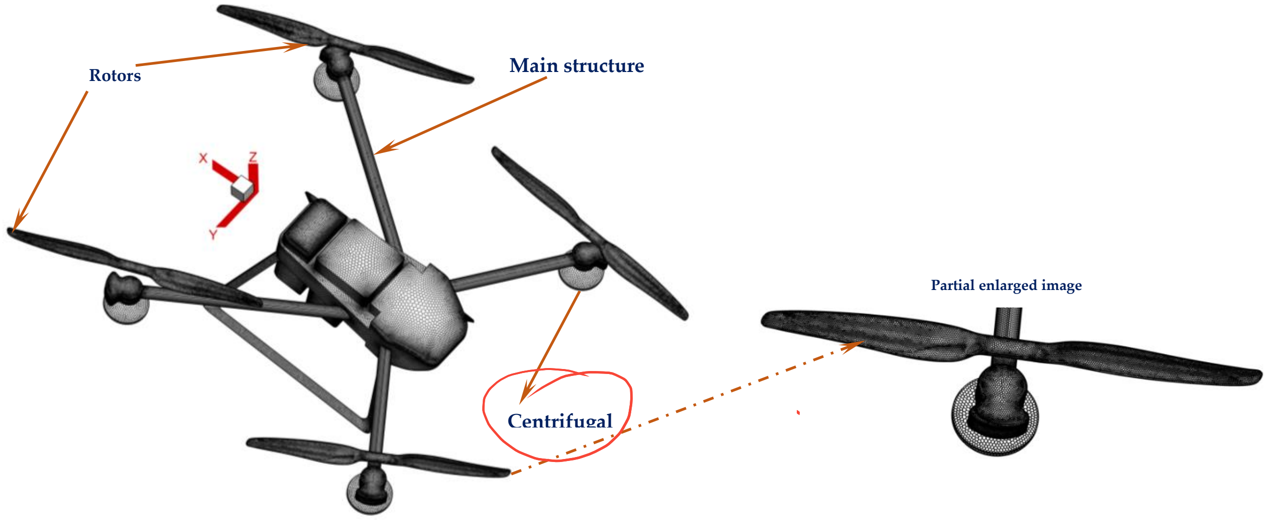

Fig 6. Grid structure diagram for the solid wall of the four-rotor UAV.

3. The evolution and distribution law of the flight-coupled airflow

3.1. *Feasibility verification of the flight-coupled airflow theoretical calculation*

To obtain a reliable dynamic distribution law of flight-coupled airflow, feasibility verification of theoretical calculation is a prerequisite before detailed analysis. In this paper, the flight-coupled wind speed measurement and calculation were strongly supported by XAIRCRAFT (Guangzhou, China). The

flight-coupled wind field testing site is shown in Fig 7a. The Bluetooth wireless wind velocity sensor (UNI-T) was applied to record the Z_b-axis wind speed values of testing points (as shown in Figs 7b and 7c), and the sampling frequency of the UNI-T sensor was 1 Hz. The test points were set right under the rotor at the UAV flight path, the height of the test points from the rotor was 2.25 m, as shown in Fig 7d. The flight direction of the four-rotor UAV was defined as the X_b-axis negative direction, the direction straight down was defined as the Z_b-axis negative direction, and the relationship between the X_b-axis, Y_b-axis, and Z_b-axis satisfied the right-hand rule, as shown in Fig 7e. The observation lines (points) for dynamic calculation of flight-coupled wind field were also illustrated in Fig 7e. According to the data provided by XAIRCRAFT, the flight speed of the four-rotor UAV was defined as 3 m/s, the forward tilt angle of the UAV body was set as 9°, the rotational angular velocity of the front two rotors was set as 249.9 rad/s, the rotational angular velocity of the rear two rotors was set as 253 rad/s. It took some time for the spatial distribution of the flight-coupled wind field to become stable after take-off, so the numerical calculations of the wind field under two working conditions were carried out. Based on working condition 1, the flow field evolution law during the transition from hover to flight state was mainly studied; the flow field distribution law after flight stabilization was analyzed in working condition 2.

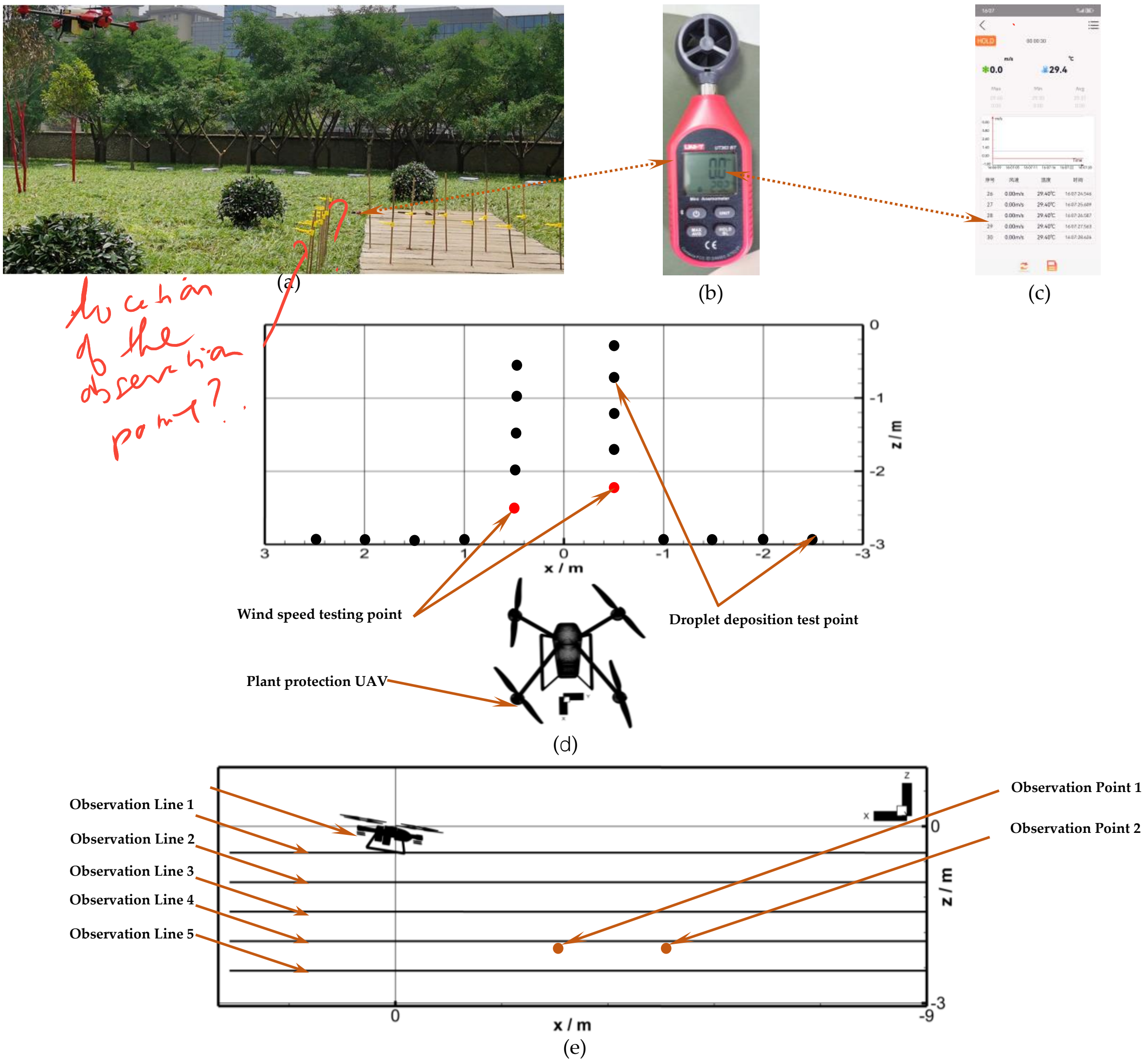

Fig 7. Wind field test and calculation observation position setting. (a) Flight-coupled wind field testing site; (b) Bluetooth wireless anemometer; (c) Bluetooth data receiving terminal; (d) Layout of measuring points of flight-coupled wind field (spray); (e) Layout diagram of observation lines (points) for dynamic calculation of flight-coupled wind field.

After the numerical calculations, the Z_b-axis velocity distribution for the longitudinal section passing through the rotor center and the Y+ cloud map of the UAV wall of the second working condition are shown in Fig 8. The Z_b-axis velocities of wind speed observation point 1 in two calculation conditions, the Z_b-axis velocities of wind speed observation point 2 in two calculation conditions, and the Z_b-axis average velocity of two test points were all presented in Fig 9.

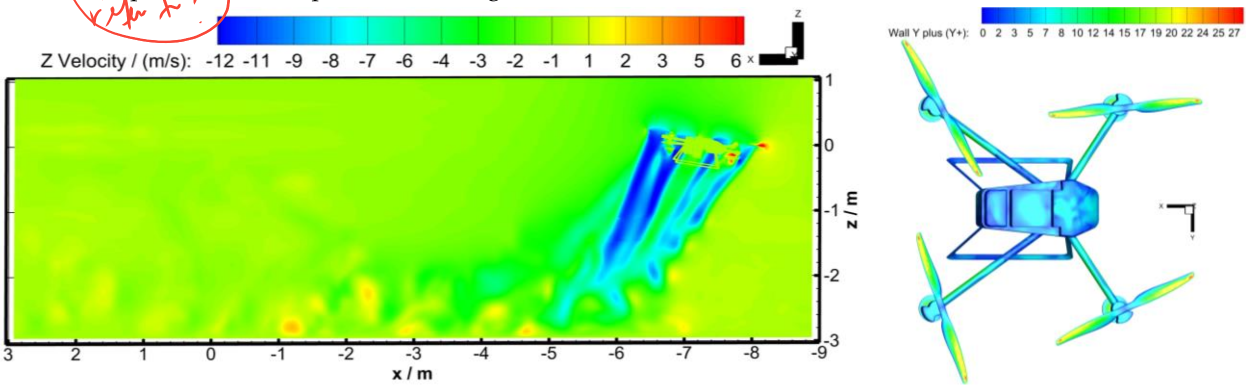

(a) (b)

Fig 8. Numerical calculation results in flight wind field. (a) Z_b-axis velocity distribution map at 2.492 s of the flight-coupled wind field for calculation condition 2; (b) Y+ sketch map for calculation condition 2 at 2.492 s.

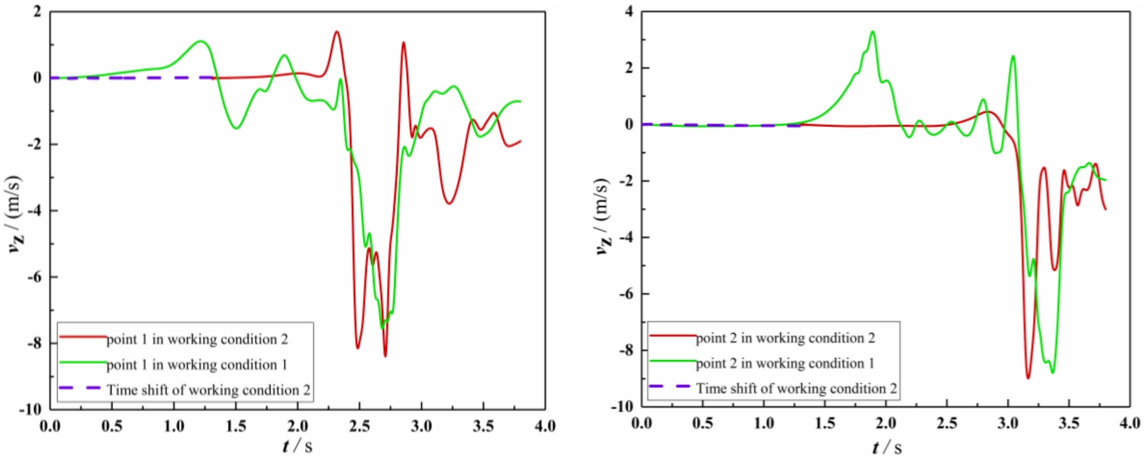

(a) (b)

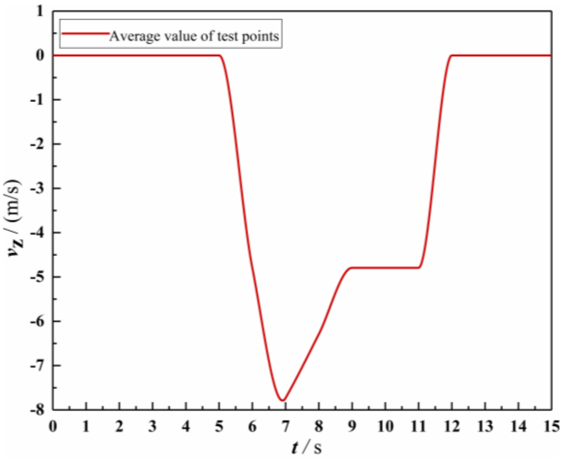

(c)

Fig 9. Comparison between numerical calculation and experiment of Z_b-axis velocity at characteristic points. (a) At feature point 1 in numerical calculations; (b) At feature point 2 in numerical calculations; (c) Average value of two test points

The calculated value of $y+$ is an important indicator of whether the thickness of the mesh boundary layer (the first layer of mesh from the solid wall to the fluid) is reasonable. When the calculated value of $y+$ is

reasonable, the development and diffusion law of the flow field can be confirmed to be reasonable. The expression for y+ is as follows [26]:

$$Y+=\frac{\Delta y\rho}{\mu}\mathrm{g}\sqrt{\frac{\tau_w}{\rho}} \tag{5}$$

Where Δy is the thickness of the first boundary layer; ρ and μ are the density and viscosity of the fluid within the first boundary layer mesh; τ_w is the shear stress on the solid wall of the first layer.

From Fig 8b, it could be seen that after the flight was in dynamic stability, the calculated value of y+ on the wall of the four-rotor plant protection UAV was below 15. Only the calculated value of y+ in the large shear area on the upper-end face of the rotor would reach 15 or more. During the flight, the calculated value of y+ in the vortex separation zone from the wing tip to one-third of the wing center would approach 30.

Taking the transient development of the flight-coupled wind field during flying, the ground effect influence on the airflow speed of the observation point, and the low sampling frequency of the UNI-T sensor into account, only the Z_b-axis velocity peaks of the testing point in wind field test and observation point in working condition 2 could be used for comparison. As could be seen from Figs 9b and 9c, the relative error of the wind speed peak in the Z_b-axis for the testing point and observation point 2 (in working condition 2) was 12.8%.

So overall, the division of the grid was reasonable.

3.2. *Evolution law of the flight-coupled airflow in spatial and temporal dimensions*

Based on the above theoretical calculation method and flight conditions, the unsteady numerical calculations of working conditions 1 and 2 were carried out. The velocity distribution diagrams of the hovering and flight wind field for working condition 1 are shown in Fig 10. In the velocity distribution diagrams, the vertical distance of the cross section from the rotor is 0.9 m, and the longitudinal section passes through the center of the rotor and is parallel to the flight direction. It could be seen that the speed of the UAV fuselage surface unit was 0 m/s when the UAV was hovering, as shown in Fig 10a. When the UAV flew forward, the speed of the UAV fuselage surface was 3 m/s, which made the hovering downwash flow in the dynamic stable state suddenly interrupted laterally, as shown in Fig 10b. It could be also seen by combining Figs 10a and 10b that the lateral displacement between the four-rotor UAV body and the airflow field occurred, and the flight wind field was thrown behind the UAV body. As could be seen from Figs 10b-10d, the downwash flow which experienced lateral interruption developed back into a dynamically stable state and eventually became the flight wind field after flying for 0.696 s. In the X_b-axis negative direction, the flight wind field was influenced by the incoming airflow from the front, then four horseshoe tails formed, rotated, and spread symmetrically backward across the cross section. It was worth mentioning that the ground effect airflow spread along the direction of flight, and the Z_b-axis velocity value at the observation point was affected to some extent by this factor. This was why working condition 2, rather than condition 1, was used to compare with the test when verifying the reliability of the numerical calculation.

The Z_b-axis velocity distribution comparison diagrams between the hover and flight phases of working condition 1 are given in Fig 11. By comparing Figs 11a-11f, it could be seen that four Z_b-axis velocity peaks appeared at observation lines 1-4 in the hovering and flight phase; the Z_b-axis velocity values of the two rotors ahead of the flight direction were smaller than the corresponding values of the two rotors behind. For the flight phase, when the vertical distance from the rotor gradually increased, the Z_b-axis wind speed peak gradually moved in the X_b-axis positive direction. During the flight phase, the dense atmosphere significantly woke the Z_b-axis wind speed peak of the front two rotors, and the Z_b-axis wind speed peak of the rear two rotors only decreased slightly. Based on Figs 10b and 11c, it could be seen that there was a significant

lateral interruption in the downwash airflow after 0.245 s of flight, and the Z_b-axis wind speed peak significantly decreased at the interruption.

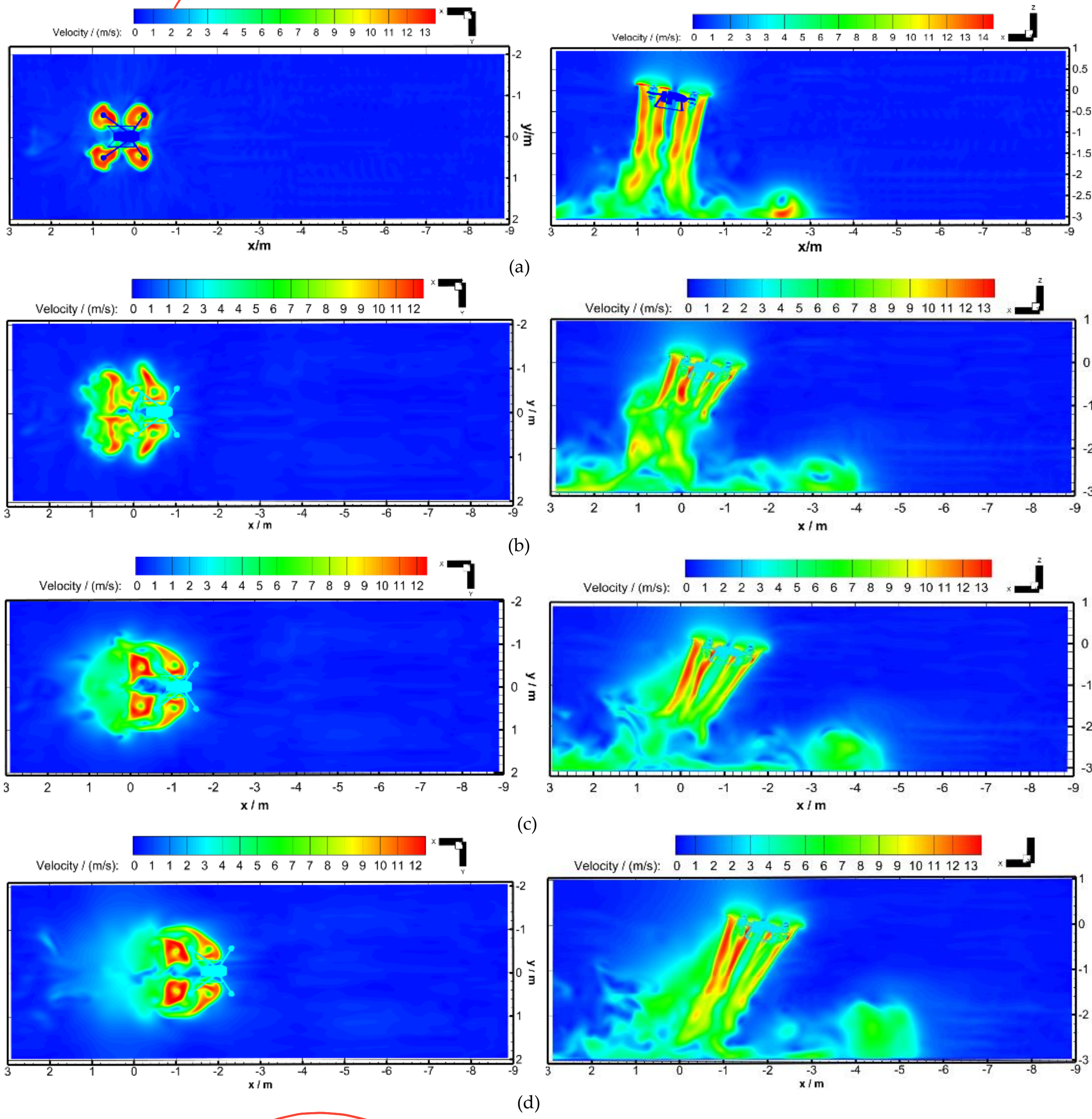

Fig 10. Horizontal and vertical section velocity distribution diagram at typical flight moments of the overall plant protection UAV (calculation condition 1). (a) After 1.3 s of hover; (b) After 0.245 s of flight; (c) After 0.446 s of flight; (d) After 0.696 s of flight.

Fig 12 shows the flow diagram of the transverse section (parallel to the horizontal direction) of the four-rotor plant protection UAV during flight. As can be seen from Figure 4, the front two rotors rotate outwards. Therefore, in forward flight, the air in front of the UAV body was compressed and flowed forward in general. Under the guidance of the front two rotors, the air near the front of the UAV body eventually moved forward and flowed to the rear side of the UAV body. When the UAV moved in the negative direction of the X_b-axis, the air density behind the body would decrease, making the air behind the UAV body flow to the front of the UAV body. Therefore, the airflow in the front direction of the UAV body and the airflow in the rear of the UAV body met at the tail of the UAV body, and formed vortex zones.

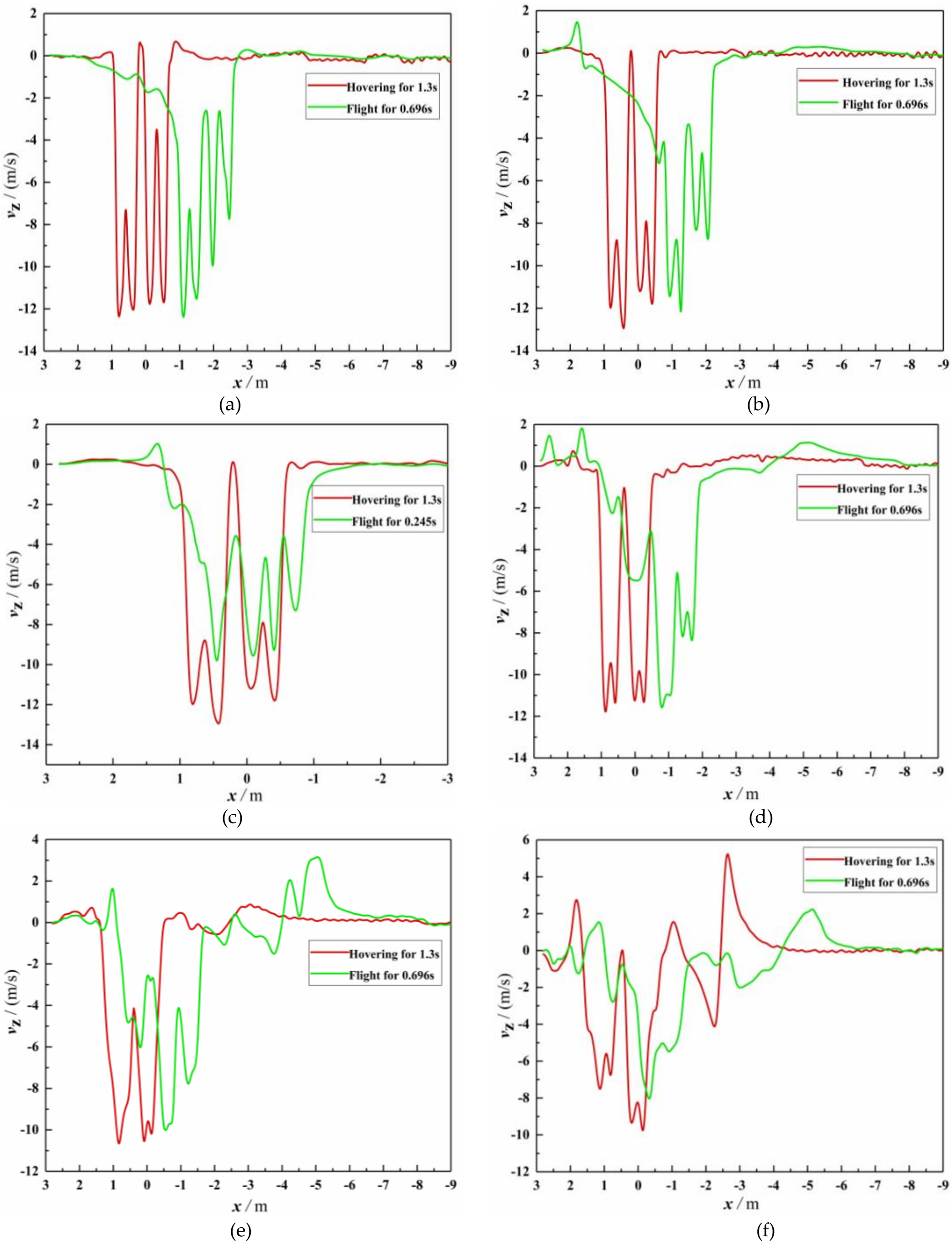

Fig 11. Comparison chart of Z_b-axis velocity on the observation line (calculation condition 1). (a) At line 1; (b) At line 2; (c) At line 2; (d) At line 3; (e) At line 4; (f) At line 5.

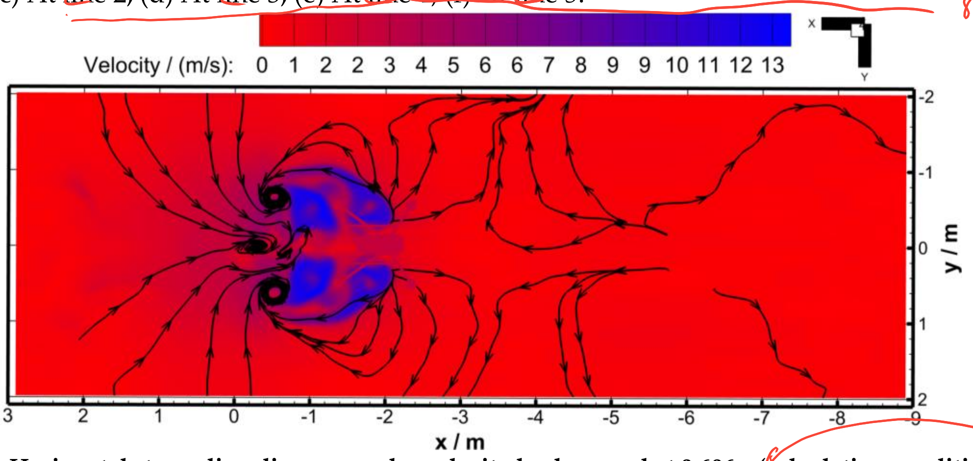

Fig 12. Horizontal streamline diagram under velocity background at 0.696 s (calculation condition 1).

In the analysis of working condition 1, it was found that the strong ground effect made numerical calculation condition 1 not suitable for verifying the reliability of numerical calculation. So the second condition that the UAV flew directly along the X_b-axis negative direction at a speed of 3m/s was executed. The velocity distribution diagrams of the cross section and longitudinal section for working condition 2 are shown in Fig 13. The Z_b-axis velocity distribution diagrams of observation lines 1 to 5 at different times in working condition 2 are also shown in Fig 14. The relative positions of the cross section, longitudinal section, and UAV were consistent with working condition 1.

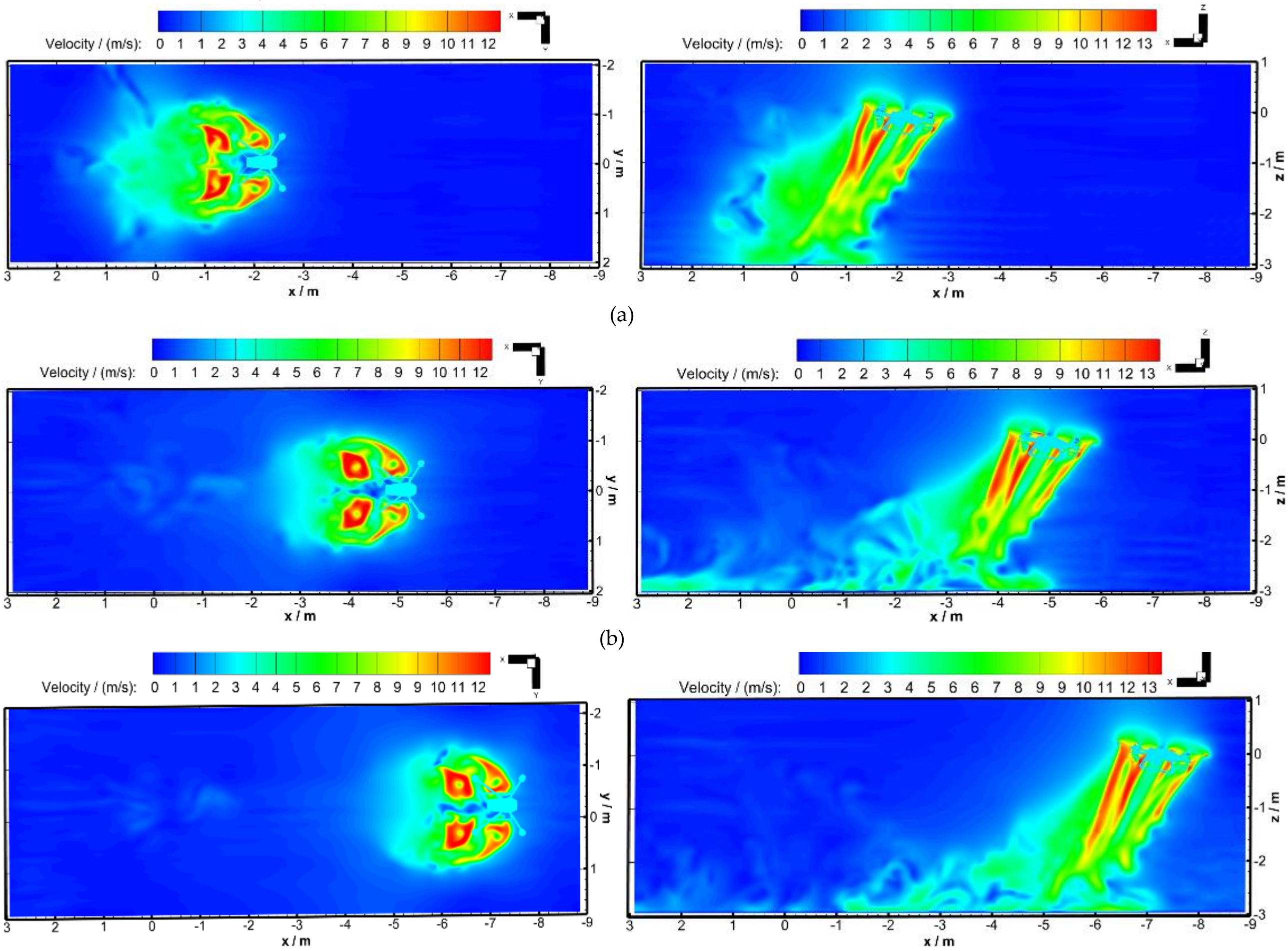

Fig 13. Horizontal and vertical section velocity distribution diagram at typical flight moments after flight (No hover process) for the overall four-rotor UAV (calculation condition 2). (a) After 0.792 s of flight; (b) After 1.742 s of flight; (c) After 2.492 s of flight.

By comparing Figs 13a-13c, it could be found that the flight-coupled wind field developed into a dynamically stable state after flying for 1.742 s. It could be seen by combining (Fig 11a, Fig 13c, and Fig 14a) that the dense atmosphere significantly woke the two flight velocity peaks of the front two rotors, and the two flight velocity peaks of the rear two rotors only decreased slightly, which led to two outcomes. The first outcome was that the absolute values of the four Z_b-axis velocity peaks from left to right along the X_b-axis negative direction at observation line 1 decreased from 12.45 m/s, 12.08 m/s, 11.77 m/s, and 11.77 m/s at hover to 11.89 m/s, 11.57 m/s, 10.71 m/s, and 8.70 m/s after the flight was stable, and the attenuation rates of Z_b-direction velocity peaks were 4.5%, 4.2%, 9.0%, and 26.1 respectively. The second outcome was that the angles between the horizontal direction and the four curves formed by Z_b-axis velocity peaks were 72°, 69°, 61°, and 56° respectively (as shown in Fig 15), but the angle values were always 90° when hovering. Therefore, to increase the effective deposition amount and improve the spraying effect, it is recommended that the centrifugal nozzle should be installed under the rear two rotors, then the angle between the horizontal direc-

tion and the centrifugal nozzle should be set between 18° and 21°. In addition, it can be seen from Fig 14 that the farther the vertical distance away from the UAV rotor, the lower the Z_b-axis wind speed value. The flight-coupled wind field would sweep over every point along the flight path, but the closer the vertical distance to the UAV rotor, the more stable the wind speed at the sweep point.

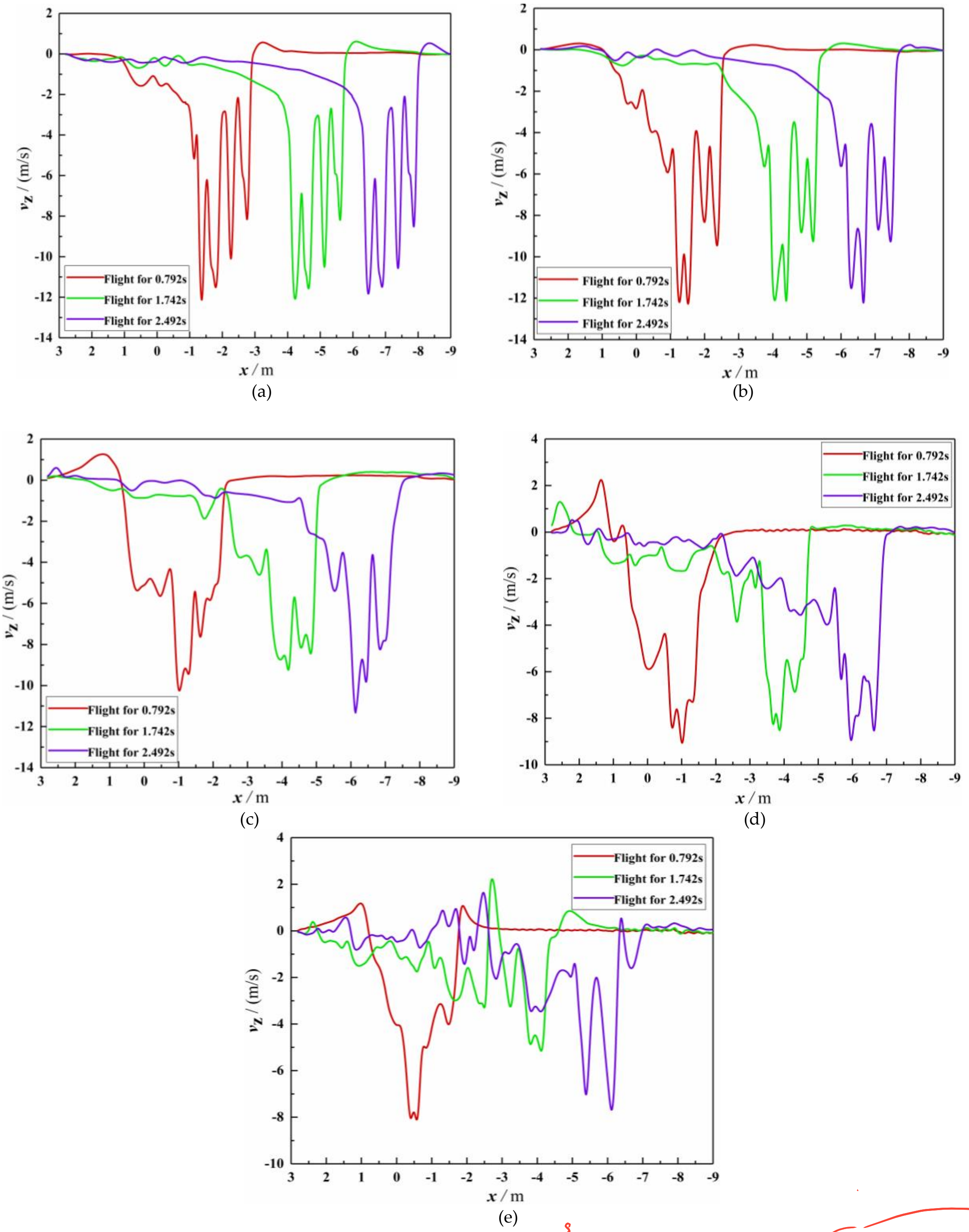

Fig 14. Comparison chart of Z_b-axis velocity on the observation line after flying for some time (calculation condition 2). (a) At line 1; (b) At line 2; (c) At line 3; (d) At line 4; (e) At line 5.

Fig 16 shows the streamline diagram of the longitudinal section (perpendicular to the horizontal direction) of the four-rotor plant protection UAV during flight. Under the pressure induced by rotor rotation, the airflow below and behind the UAV body flowed downward as a whole. The UAV had a forward inclination

of 9° and the ground was a non-slip wall boundary. Under the guidance of the downwash that developed diagonally backward, the airflow flowing down would eventually develop toward the rear. The downwash airflow that developed diagonally backward had a high speed and could fully develop to the ground, forming a diagonally downward "wind wall". So in the process of flying forward, this "wind wall" will drive the air in front of the UAV body to continue to flow forward.

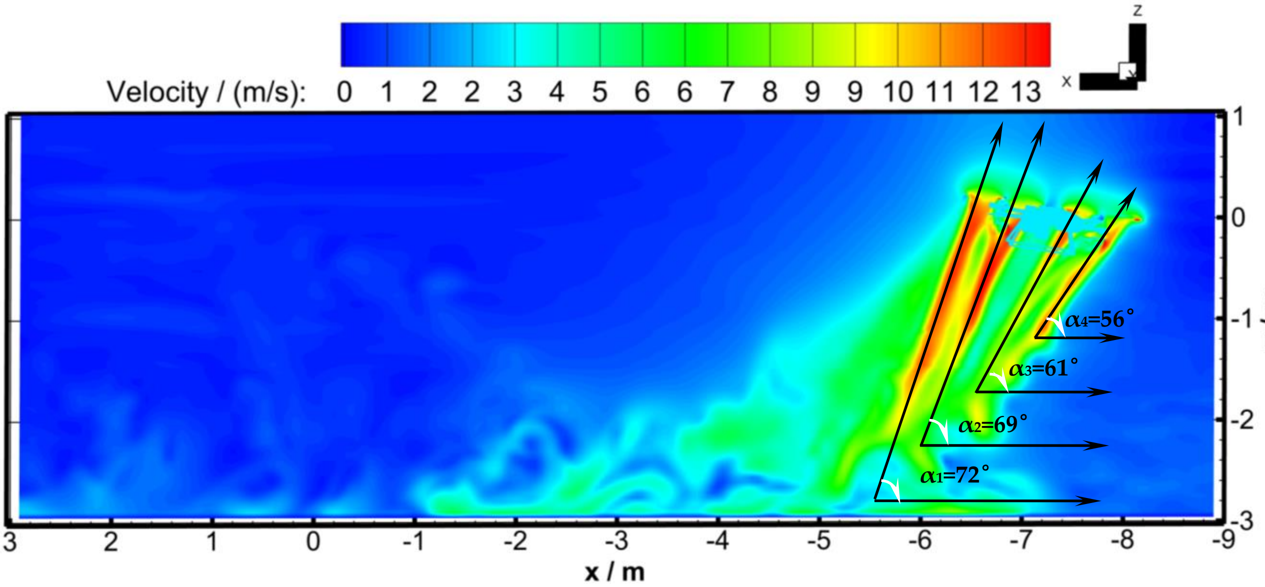

Fig 15. The angles between the horizontal direction and the four curves formed by Z_b-direction velocity peaks

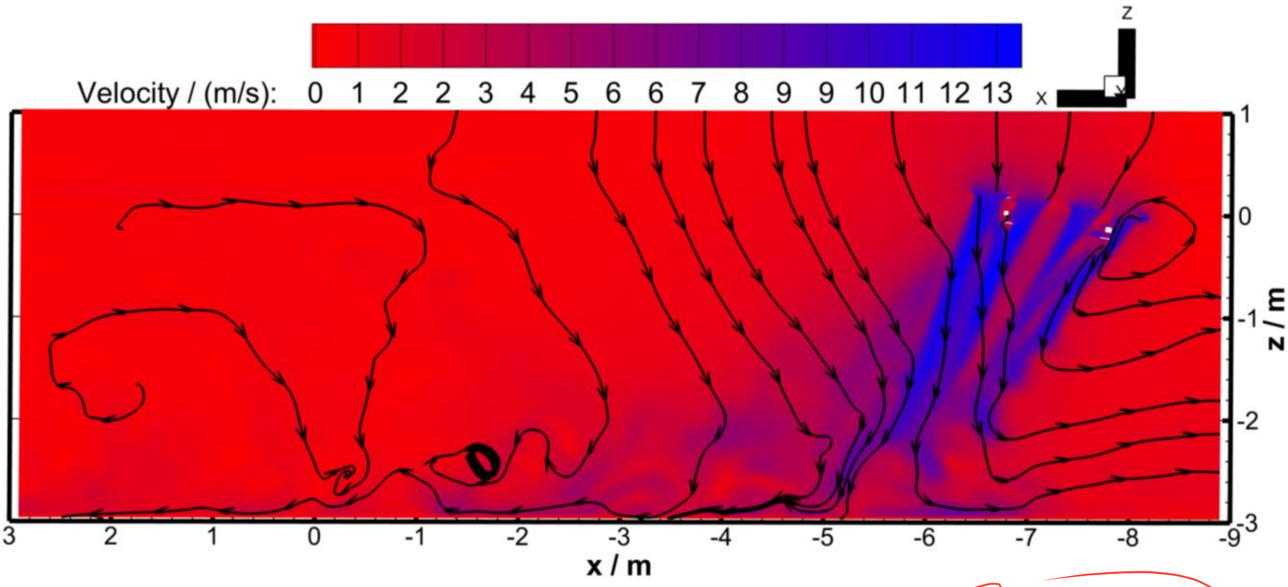

Fig 16. longitudinal streamline diagram under velocity background at 2.492 s (calculation condition 2).

4. Conclusions

This research was motivated by a deep understanding of flight-coupled airflow spatiotemporal evolution law for the four-rotor plant protection UAV. The flight-coupled airflow simulation and the wind speed testing based on the overall UAV were performed, and the conclusions were drawn:

(1) Two calculation conditions were performed to analyze the evolution of the flight-coupled wind field. Condition 1 corresponded to the flow field distribution law study of hover and flight state switching, and Condition 2 corresponded to the flow field distribution law study in the flight state. The Z_b-axis wind speed value of the observation point in condition 2 was used to compare with the wind value of the testing point,

and the relative error was 12.8%. The analysis of the flight-coupled airflow based on the numerical calculation was reliable.

(2) When switching from hover to flight state in calculation condition 1, the downwash wind field experienced a lateral interruption and developed into the stable flight-coupled wind field after flying for 0.696 s. Therefore, the nozzles were recommended to open and spray until the flight-coupled wind field developed into a stable flow, then the massive droplet drift caused by turbulent wind flow during the initial stage of flight would be significantly reduced.

(3) For the four-rotor UAV, the dense atmosphere reduced the absolute values of the four Z_b-axis velocity peaks from left to right along the flight direction by 4.5%, 4.2%, 9.0%, and 26.1% respectively at observation line 1. The angles between the horizontal direction and the four curves formed by Z_b-axis velocity peaks were also changed from 90° to 72°, 69°, 61°, and 56° respectively. Therefore, to enhance the downward transport effect of the flight-coupled wind field on the droplets, and ultimately improve the deposition effect of the canopy, the nozzles were proposed to be installed under the rear two rotors, and the angle between the horizontal direction and centrifugal nozzle (perpendicular to the flight-coupled wind field) should be set between 18° and 21° when the flight speed was 3m/s.

5. Future Work

The research in this paper can provide a guide for the research on setting the flight buffer distance at different flight speeds before opening the spray nozzle in the future. This work can also lay a foundation for the mechanism study of flight airflow-pesticide droplet-crop canopy interaction for the multi-rotor plant protection UAV.

Acknowledgments: The authors acknowledge the support provided by the National Natural Science Foundation of China (No. 52275257, No. 51705264).

Author Contributions: Yang Fengbo completed the theoretical calculation. Li Zhiwei designed the experiments. Yang Fengbo wrote the paper. Zhang Guangyao and Zhou Hongping completed the English revisions. The authors have read and agreed to the final version of the manuscript.

Conflicts of Interest: The authors declare no conflict of interest.

References

1. Lan YB, Chen SD, Fritz BK. Current status and future trends of precision agricultural aviation technologies. International. J. Agric. Biol. Eng. 2017, 10(3): 1-17.
2. Li JY, Lan YB, Shi Y. Research progress on airflow characteristics and field pesticide application system of rotary-wing UAV. Trans. Chin. Soc. Agric. Eng. 2018, 34(12): 104-118.
3. Guo QW, Zhu YZ, Tang Y, Hou CJ, He Y, Zhuang JJ, Zheng YL, Luo SM. CFD simulation and experimental verification of the spatial and temporal distributions of the downwash airflow of a quad-rotor agricultural UAV in hover. Comput. Electron. Agric. 172 (2020), 105343.
4. Zhang S, Huang M, Cai C, Sun H, Cheng X, Fu J, Xing Q, Xue X. Parameter Optimization and Impacts on Oilseed Rape (Brassica napus) Seeds Aerial Seeding Based on Unmanned Agricultural Aerial System. Drones, 2022, 6, 303.
5. Qin WC, Qiu BJ, Xue XY, Chen C, Xu ZF, Zhou QQ. Droplet deposition and control effect of insecticides sprayed with an unmanned aerial vehicle against plant hoppers. Crop Prot. 2016; 85: 79-88.
6. Yang FB, Xue XY, Zhang L, Sun Z. Numerical simulation and experimental verification on downwash air flow of six-rotor agricultural unmanned aerial vehicle in hover. Int J Agric & Biol Eng, 2017; 10(4): 41-53.
7. Zhang YC, Li YJ, He Y, Liu F, Cen HY, Fang H. Near ground platform development to simulate UAV aerial spraying and its spraying test under different conditions. Comput. Electron. Agric. 148(2018): 8-18.

8. Zhu YZ, Guo QW, Tang Y, Zhu X, He Y, Huang HS, Luo SM. CFD simulation and measurement of the downwash airflow of a quadrotor plant protection UAV during operation. Comput. Electron. Agric. 201(2022), 107286.
9. Li JY, Shi YY, Lan YB, Guo S. Vertical distribution and vortex structure of rotor wind field under the influence of rice canopy. Comput. Electron. Agric. 2019, 159: 140-146.
10. Tang Q, Zhang RR, Chen LP, Yi TC, Zhang B. Droplets movement and deposition of an eight-rotor agricultural UAV in downwash flow field. Int J Agric & Biol Eng. 2017; 10(3): 47-56.
11. Tian ZW, Xue XY, Xu Y, Yang FB, Sun Z. Effect of Plant Protection UAVs Downwash on Crop Canopy. Trans. Chin. Soc. Agric. Mach. 2021, 52(1): 40-48.
12. Chen SD, Lan YB, BRADLEY KF, LI JY, Liu AM, Mao YD. Effect of Wind Field below Rotor on Distribution of Aerial Spraying Droplet Deposition by Using Multi-rotor UAV. Trans. Chin. Soc. Agric. Mach. 2017, 48(8): 105-113.
13. Zhang B, Tang Q, Chen LP, Xu M. Numerical simulation of wake vortices of crop spraying aircraft close to the ground. Biosyst. Eng. 2016, 145, 52-64.
14. Zhang B, Tang Q, Chen LP, Zhang RR, Xu M. Numerical simulation of spray drift and deposition from a crop spraying aircraft using a CFD approach. Biosyst. Eng. 2018, 166, 184-199.
15. Jung YS, You JY, Kwon OJ. Numerical investigation of prop-rotor and tailwing aerodynamic interference for a tilt-rotor UAV configuration. Mech. Sci. Technol. 2014, 28 (7), 2609-2617.
16. Zhang H, Qi LJ, Wu YL, Liu WW, Musiu E, Xiao Y, Yang ZP. Research on spatio-temporal distribution of down-wash airflow for multi-rotor plant protection UAV based on porous model. Trans. Chin. Soc. Agric. Mach. 2018, 50(02), 112-122.
17. Li JY, Shi YY, Lan YB, Guo S. Vertical distribution and vortex structure of rotor wind field under the influence of rice canopy. Comput. Electron. Agric. 2019, 159, 140-146.
18. Wang CL, He XK, Wang XN, Wang ZC, Wang SL, Li LL, Bonds J, Herbst A, Wang ZG. Testing method and distribution characteristics of spatial pesticide spraying deposition quality balance for unmanned aerial vehicle. Int J Agric & Biol Eng. 2018, 11(2), 18-26.
19. Wang P, Hu L, Zhou ZY, Yang WS, Liu AM, Luo XW, Xue XY, He J, Yan Y. Wind field measurement for supplementary pollination in hybrid rice breeding using unmanned gasoline engine single-rotor helicopter. Trans. Chin. Soc. Agric. Eng. 2013, 29(3), 54-61.
20. Li JY, Zhou ZY, Lan YB, Hu L, Zhang Y, Liu AM, Luo XW, Zhang TM. Distribution of canopy wind field produced by rotor unmanned aerial vehicle pollination operation. Trans. Chin. Soc. Agric. Eng. 2015, 31 (3), 77-86.
21. Endalew AM, Debaer C, Rutten N, Vercammen J, Delele MA, Ramon H, Nicolaï BM, Verbovena P. Modelling pesticide flow and deposition from air-assisted orchard spraying in orchards: A new integrated CFD approach. Agric. Forest Meteorol. 2010, 150, 1383–1392.
22. S`alcedo R, Vallet A, Granell R, Garcerá C, Moltó E, Chueca P. Euleriane Lagrangian model of the behaviour of droplets produced by an air-assisted sprayer in a citrus orchard. Biosyst. Eng. 2017, 154, 76–91.
23. Agarwal RK, Deese JE. Euler calculations for flow field of a helicopter rotor in hover. J. Aircraft. 1987, 24(4), 231–238.
24. Gao XF, Shi WD, Zhang DS, Zhang QH, Fang B. Optimization Design and Test of Vortex Pump Based on CFD Orthogonal Test. Trans. Chin. Soc. Agric. Mach. 2014, 45, 101–106.
25. Niu YS. Research on Internal Ballistic Flow Filed Characteristics of Self-Eject Launch. Beijing, Beijing Institute of Technology, 2016.
26. Roberts TW, Murman, EM. Solution method for a hovering helicopter rotor using the Euler equations. In Proceedings of the 23rd Aerospace Sciences Meeting, Reno, NV, USA, 14–17 January 1985.
