# Supplementary material for: The spatiotemporal evolution of flight-coupled wind field for a four-rotor plant protection unmanned aerial vehicle
Source: PLoS One. 2025 May 8;20(5):e0318769. doi: 10.1371/journal.pone.0318769 (PMC12061431; doi:10.1371/journal.pone.0318769)
Supplement: Attachment — Submitted filename: Response to Reviewers.docx [file pone.0318769.s003.docx]

**Dear Editor, Dear reviewers,**

Thank you for your letter dated July 29th. We thank you very much for the time and effort that they have put into reviewing the previous version of the manuscript. The suggestions have enabled us to improve our work. This document is our point-by-point response to the comments raised by the reviewers. The comments are reproduced and our responses are given directly afterward in a different color . We would like also to thank you very much for allowing us to resubmit a revised copy of the manuscript.

**Reviewer's Responses to Questions**

1. Is the manuscript technically sound, and do the data support the conclusions?

The manuscript must describe a technically sound piece of scientific research with data that supports the conclusions. Experiments must have been conducted rigorously, with appropriate controls, replication, and sample sizes. The conclusions must be drawn appropriately based on the data presented.

**Reviewer #1: Yes	Reviewer #2: No**

**The authors' reply：**Thank you very much for the professional and detailed review comment from the reviewer. Thanks to the first reviewer for the affirmation and encouragement. As the second reviewer pointed out in the specific comments, the details of model constructions, calculation methods, theoretical and experimental comparisons were not fully discussed in the first edition of the paper, and it made the reproducibility of the paper appear insufficient. Dear reviewer, in order to make the theoretical calculation part of the paper have sufficient reproducibility, the Sections 2 and 3.1 of the modified version had been expanded in detail. Please refer to sections 2.3(Structure, Boundary Condition, and Mesh Model), 2.4(Numerical Solution Method), and 3.1(Feasibility Verification of the Flight-Coupled Airflow Theoretical Simulation) in the revised version for details.

2. Has the statistical analysis been performed appropriately and rigorously?

**Reviewer #1: Yes	Reviewer #2: N/A**

**The authors' reply：**Thank you very much for the professional and detailed review comment from the reviewer. Thanks to the first reviewer for the affirmation and encouragement. As the second reviewer pointed out “I don't know which are the calculation results and which are the experimental results. Are they really compared ? ” in the specific comments. To solve this problem, Sections 2.3 and 3.1 have been modified in detail. Then the layout of the test, the acquisition of experimental data, the detailed method and process of theoretical calculation, the comparison between the results of the test and theoretical calculation appeared clearer, and the analysis of the data also appeared more rigorous.

3. Have the authors made all data underlying the findings in their manuscript fully available?

The [PLOS Data policy](http://www.plosone.org/static/policies.action#sharing) requires authors to make all data underlying the findings described in their manuscript fully available without restriction, with rare exception (please refer to the Data Availability Statement in the manuscript PDF file). The data should be provided as part of the manuscript or its supporting information, or deposited to a public repository. For example, in addition to summary statistics, the data points behind means, medians and variance measures should be available. If there are restrictions on publicly sharing data-e.g. participant privacy or use of data from a third party—those must be specified.

**Reviewer #1: Yes	Reviewer #2: No**

**The authors' reply：**Thank you very much for the professional and detailed review comment from the reviewer. Thanks to the first reviewer for the affirmation and encouragement . As the second reviewer pointed out “What grid generation software is used? If the code is in-house, specific schemes should be explained, or published papers should be cited”, “What software is being used for the NS solver as well? In the case of in-house code, specific calculation methods, time integration methods, pressure coupling, etc. should be explained”, “I don't know which are the calculation results and which are the experimental results. Are they really compared? ”, and “It is necessary to explain what kind of boundary conditions are imposed on the pressure and velocity by using specific mathematical expressions” in the specific comments.

In response to these problems, the grid generation software and detailed data of the grid model, the N-S equation solution software and solution method data, the boundary conditions and its specific mathematical expressions, the results of the test and theoretical calculation were all reorganized and presented in detail in the modified version.

4. Is the manuscript presented in an intelligible fashion and written in standard English?

PLOS ONE does not copyedit accepted manuscripts, so the language in submitted articles must be clear, correct, and unambiguous. Any typographical or grammatical errors should be corrected at revision, so please note any specific errors here. **Reviewer #1: Yes Reviewer #2: No**

**The authors' reply：**Thank you very much for the professional and detailed review comment from the reviewer. Thanks to the first reviewer for the affirmation and encouragement. In response to the suggestions made by two reviewers to improve some of the wordings and phrases and also the format of the paper, all modifications have been made in the revised version.

**Review Comments to the Author**

**Reviewer #1:**

**1. Having read the article, I am a little confused on the difference in the terms Working Conditions, Conditions and Calculation Conditions 1 and 2? Do they refer to the same thing?**

**The authors' reply：**Thank you very much for the professional and detailed review comment from the reviewer. Dear reviewer, the expressions "Working Conditions", "Conditions" and "Calculation Conditions" refer to the same thing. As the reviewer pointed out, when writing the paper in English, the authors expressed the “two conditions of theoretical calculation” in various English forms, which caused ambiguity. Therefore, these expressions were uniformly modified as "Working Condition" in the revised version of the manuscript. All the modified parts in the revised manuscript were marked in red.

**2.The Equation 1 and 2, I suggest that they come one after the other and not separated by Fig. 2 as the definitions of the symbols are all given in the same paragraph after Equation 2. Fig. 2 should be after the last paragraph of the Introduction section.**

**The authors' reply：**Thank you very much for the professional and detailed review comment from the reviewer. As the reviewer pointed out, when writing the paper in English, Fig 2 was placed after the third page to make the layout look better, and then it led to an unreasonable circumstance between Equations 1 and 2. Therefore, Fig. 2 was placed in the last paragraph of the Introduction Section in the revised version.

**3. For Table 1, maybe the columns can be separated into 3 representing *x*, *y* and *z*.**

**The authors' reply：**Thank you very much for the professional and detailed review comment from the reviewer. According to the opinion of the reviewer, Table 1 was broken down into Table 1 and Table 2, and the forms of the two tables were redesigned. The revised Table 1 and Table 2 were shown below. All the modified parts in the revised version were marked in red.

**Table 1. Running parameters of each rotor in Working Condition** 1

| Rotor number | Coordinates of the rotation center  in Working Condition 1 | | | | Angular velocity (rad/s) |
| --- | --- | --- | --- | --- | --- |
|  | *x /* m | | *y /* m | *z /* m |  |
|  | *t*≤1.3(s)（UAV Fixed） | *t*＞1.3(s)（UAV Flight） |  |  |  |
| 1 | -0.415239839 | -0.415239839-(3*t*-3.9) | -0.518860346 | -0.099685111 | 249.9 |
| 2 | -0.415239839 | -0.415239839-(3*t*- 3.9) | 0.518860347 | -0.099685111 | -249.9 |
| 3 | 0.541537512 | 0.541537512-(3*t*- 3.9) | 0.516302113 | 0.051853534 | 253 |
| 4 | 0.541537512 | 0.541537512-(3*t*- 3.9) | -0.516302112 | 0.051853534 | -253 |

**Table 2. Running parameters of each rotor in Working Condition** 2

| Rotor number | Coordinates of the rotation center  in Working Condition 2 | | | Angular velocity (rad/s) |
| --- | --- | --- | --- | --- |
|  | *x /* m （UAV Flight） | *y /* m | *z /* m |  |
| 1 | -0.415239839-3*t* | -0.518860346 | -0.099685111 | 249.9 |
| 2 | -0.415239839-3*t* | 0.518860347 | -0.099685111 | -249.9 |
| 3 | 0.541537512-3*t* | 0.516302113 | 0.051853534 | 253 |
| 4 | 0.541537512-3*t* | -0.516302112 | 0.051853534 | -253 |

**4. For Fig. 5, does the location of the computational domain @ Domain 1 - Domain 4 correspond to the Interface 1-4?**

**The authors' reply：**Thank you very much for the professional and detailed review comment from the reviewer. Dear reviewer, the rotor of the UAV rotated while the UAV body tilted forward by 9 ° and flew at a speed of 3m/s along the negative *x*-axis direction. As the reviewer pointed out, in order to achieve dynamic calculation, the calculation area was divided into 4 domains (as shown in the figure below). The rules of movement were different in different regions. An interface was formed between overlapping surfaces of two adjacent regions. Real-time exchanges of flow field data were realized between the overlapping surfaces in adjacent regions through the interface.

So from the figure below, it could be seen that Computing Domain 1 and Computing Domain 2 were connected and flow field data were exchanged through Interface 1, Computing Domain 2 and Computing Domain 3 were connected and flow field data were exchanged through Interface 2, Computing Domain 3 and Computing Domain 4 were connected and flow field data were exchanged through Interface 3. In accordance with the opinion of the reviewer, Fig 5 had also been adapted in the revised version. (Therefore, the model constructed in this paper contained four domains and three interfaces)

**Fig 5.** **Calculating regions connections and boundary surfaces markings**

**5. Is testing point, observation and feature point the same?**

**The authors' reply：**Thank you very much for the professional and detailed review comment from the reviewer. Dear reviewer, the expressions "Testing Point", "Observation Point" and "Feature point " are not the same. In order to better explain the difference between them, the following figures 7(d) and 7(e) were drawn to illustrate. “Testing Point” referred to the point at which anemometer was used to record vertical wind speed during flight wind field test, as shown in Fig 7(d) below. “Observation Point” refers to the point at which vertical wind speed was observed through the calculation window during the numerical calculation of flight-coupled wind field, as shown in Fig 7(e) below. “Feature point” was a less relevant expression when writing the first draft, this expression had been replaced by “Observation Point” in the Introduction of the revised version. In addition, to better illustrate the difference between "Testing Point" and "Observation Point", Table 4 had been added to Section 3.1. All the modified parts in the revised version were marked in red.

**(d)**

**(e)**

**Fig 7.** **Wind field test and calculation observation position setting.** …...(d) Layout of testing points of flight-coupled wind field (spray); (e) Layout diagram of observation lines (points) for dynamic calculation of flight-coupled wind field.

**Table 4. Comparison of peak vertical velocity values between test points and observation points**

| Object | | Coordinates | | Peak vertical  velocity | | Coordinates | | | Peak vertical velocity | Absolute value of peak velocity relative error |
| --- | --- | --- | --- | --- | --- | --- | --- | --- | --- | --- |
|  |  | *y /* m | *z /* m | *V*_Z_-Peak */* (m/s) | Average value  */* (m/s) | *x /* m | *y /* m | *z /* m | *V*_Z_-Peak  */* (m/s) | Relative error  */* % |
| Wind field test | Testing Point 1 | -0.516302 | -2.25 | -8.0 | -7.9 | \ | \ | \ | \ | \ |
|  | Testing Point 2 | 0.516302 | -2.25 | -7.7 |  | \ | \ | \ | \ | \ |
| Calculation Working Condition 1 | Observation Point 1 | \ | \ | \ | \ | -2.536297 | -0.516302 | -2.25 | -7.5 | 5.1 |
|  | Observation Point 2 | \ | \ | \ | \ | -4.536297 | -0.516302 | -2.25 | -8.7 | 10.1 |
| Wind field test | Testing Point 1 | -0.516302 | -2.25 | -8.0 | -7.9 | \ | \ | \ | \ | \ |
|  | Testing Point 2 | 0.516302 | -2.25 | -7.7 |  | \ | \ | \ | \ | \ |
| Calculation Working Condition 2 | Observation Point 1 | \ | \ | \ | \ | -2.536297 | -0.516302 | -2.25 | -8.4 | 6.3 |
|  | Observation Point 2 | \ | \ | \ | \ | -4.536297 | -0.516302 | -2.25 | -8.9 | 12.7 |

**6. Maybe can explain more on what is the flow field evolution law mentioned.**

**The authors' reply：**Thank you very much for the professional and detailed review comment from the reviewer. Dear reviewer, the numerical calculations performed in this paper were transient working conditions, so the time of each calculation result figure was given. These cloud figures and velocity distribution figures at a certain time had be analyzed in this paper. The description may focus too much on a certain state, the changes and connections between the states appeared to be lacking. Therefore, the description of spatiotemporal evolution of Flight-Coupled Wind Field was appropriately added at Section 3.2, and a systematic description of flow evolution was carried out at the conclusion part in the revised version (as shown in the following paragraph). All the modified parts in the revised version were marked in red.

(2) In the simulation Working Conditions 1 and 2, the evolution of flight-coupled wind field for the four-rotor Plant Protection UAV had undergone a dynamic process. During the process from hover to flight, the “downwash airflow” experienced lateral interruption, developed back into a dynamically stable state and eventually became the “flight-coupled wind field” after flying for 0.696 s. During the process from hover to flight, the “flight-coupled wind field” distribution area of the cross section developed from directly below the UAV body to gradually behind the UAV body, and finally maintainde a stable transverse distance from the UAV body. During the process from hover to flight, the “flight-coupled wind field” was influenced by the incoming airflow from the front, the four circular wind fields on the cross section developed into four horseshoe tails, which rotated, and spread symmetrically backward across the cross section in the *X*_b_-axis negative direction. During the process from hover to flight, the “flight-coupled wind field” in the longitudinal section developed from relatively stable to the phenomenon that vortices constantly fell off in the opposite direction of flight.

**7. Fig. 6 - the bottom part of the Centrifugal wording seems to be missing.**

**The authors' reply：**Thank you very much for the professional and detailed review comment form the reviewer. In accordance with the opinion of the reviewer, Fig 6 had been modified, and the change was marked in red in the revised version, as shown the following figure.

**Fig 6. Grid structure diagram for the solid wall of the four-rotor UAV.**

**8. Maybe can use another better wording for the phrase 'dug out' in line 190.**

**The authors' reply：**Thank you very much for **the professional and detailed review comment** from the reviewer. The phrase 'dug out' appeared twice in the initial version. In accordance with the opinion of the reviewer, “deleted” was used instead of 'dug out' in the revised version. And the changes were marked in red at Section 2.3.2(2) in the revised version.

**9. For equation 4 , maybe can define each of the symbol used.**

**The authors' reply：**Thank you very much for the professional and detailed review comment from the reviewer. When writing the manuscript, some variables were not defined due to the author's carelessness. All variables in equation 4 had been defined in the revised version, as shown in the following statement. And the changes were marked in red in the revised version.

*k* equation:

$\frac{\partial\left(\rho k\right)}{\partial t}+\frac{\partial\left(\rho ku_{i}\right)}{\partial x_{i}}=\frac{\partial}{\partial x_{j}}\left[\alpha_{k}\mu_{eff}\frac{\partial k}{\partial x_{j}}\right]+G_{k}+G_{b}-\rho\varepsilon$ (3)

*ε* equation:

$\frac{\partial\left(\rho\varepsilon\right)}{\partial t}+\frac{\partial\left(\rho\varepsilon u_{i}\right)}{\partial x_{i}}=\frac{\partial}{\partial x_{j}}\left[\alpha_{\varepsilon}\mu_{eff}\frac{\partial\varepsilon}{\partial x_{j}}\right]+C^{*}_{1\varepsilon}\frac{\varepsilon}{k}G_{k}-C_{2\varepsilon}\rho\frac{\varepsilon^{2}}{k}$ (4)

$C_{1\varepsilon}^{*}=C_{1\varepsilon}-\frac{\eta\left(1-\eta/\eta_{0}\right)}{1+\beta\eta^{3}}$ (5)

 (6)

$E_{ij}=\frac{1}{2}\left(\frac{\partial u_{i}}{\partial x_{j}}+\frac{\partial u_{j}}{\partial x_{i}}\right)$ (7)

**Where** *ρ* is the airflow density of a mesh unit; *k* and *ε* are the turbulent kinetic energy and the turbulent dissipation rate; *t* is the current calculation time; $u_{i}$is the time mean velocity in the direction *i*; $x_{i}$and$x_{j}$ are coordinates in the *i* and *j* directions; $\alpha_{k}$and$\alpha_{\varepsilon}$ are turbulence model constants,$\alpha_{k}=\alpha_{\varepsilon}=1.39$; $\mu_{eff}$is total viscosity of airflow, $\mu$is kinematic viscosity of airflow, $\mu_{t}$is turbulent viscosity of airflow, $\mu_{eff}=\mu+\mu_{t}$,$\mu_{t}=\rho C_{\mu}\frac{k^{2}}{\varepsilon}$; $G_{k}$ is the production term of turbulent kinetic energy *k* due to the average velocity gradient; $G_{b}$is the production term of turbulent kinetic energy *k* caused by buoyancy; $C_{1\varepsilon}^{*}$is an incremental modification of the RNG *k*-*ε* equation based on the Standard *k*-*ε* equation; $\eta$is the ratio of turbulence to mean flow in time scale; $E_{ij}$is characteristic strain rate; $C_{\mu}$,$C_{1\varepsilon}$,$\eta_{0}$,$\beta$,$C_{2\varepsilon}$ are Constant terms, $C_{\mu}=0.0845$,$C_{1\varepsilon}=1.42$,$\eta_{0}=4.377$,$\beta=0.012$,$C_{2\varepsilon}=1.68$.

**10. There are a few suggestions to improve some of the wordings and phrases and also the format of the paper. I have submitted my review of the paper for the author's reference.**

**The authors' reply:** Thank you very much to the reviewer for your professional and detailed review of the paper. According to the revision clues indicated by the reviewer in the paper, we made all revisions. In addition, the revision clues marked by the reviewer would lead to some related areas that needed to be refined or modified, and we had also made modifications. And the changes were marked in red in the revised version.

**Reviewer #2:** **Overall, the description of the numerical method is insufficient, and there is no reproducibility.**

Dear reviewer, thank you very much for the professional review comment from you. In order to make the theoretical calculation part of the paper have sufficient reproducibility, the Sections 2 and 3.1 of the modified version had been expanded in detail. Please refer to sections 2.3(Structure, Boundary Condition, and Mesh Model), 2.4(Numerical Solution Method), and 3.1(Feasibility Verification of the Flight-Coupled Airflow Theoretical Simulation) in the revised version for details.

The following text is the detailed revision and reply to the specific questions of the reviewer.

**1. What is the Z_b_ direction? Since there is no explanation, the meaning is unclear within the abstract.**

**The authors' reply：**Thank you very much for the professional and detailed review comment from the reviewer. Dear reviewer, the motion system diagram of the four-rotor plant protection UAV is shown in the following Fig 4. There were two kinds of coordinate systems in Fig 4, namely the absolute coordinate system *O*_b_*X*_b_*Y*_b_*Z*_b_ and the relative coordinate system *O*_e_*X*_e_*Y*_e_*Z*_e_; and the coordinate systems were set according to the right-hand rule. The geometric center of each rotor was the coordinate origin of the respective relative coordinate system. The absolute coordinate system was used to set the position and rotation angular speed of each rotor, and the relative coordinate system was used to set the rotation axis of each rotor. This description was added and marked in red at Section 2.3.2(1) in the revised version.

Therefore, dear reviewer, Z_b_ is the Z direction in the absolute coordinate system. In the revised version, Z_b_ was also explained in the abstract.

**Fig 4. Four-rotor UAV motion system diagram.**

**2. The symbol list should be added.**

**The authors' reply：**Thank you very much for the valuable and detailed review comment from the reviewer. As pointed out by the reviewer, there were too many variables and abbreviations in the paper. In order to increase the readability of the paper, a “Nomenclature” table was added at the beginning of the revised version, as shown below.

| **Nomenclature** | |
| --- | --- |
| **Abbreviations** | |
| UAV | Unmanned Aerial Vehicle |
| CFD | Computational Fluid Dynamics |
| RANS | Reynolds Averaged Naviere-Stokes |
| RNG | Re-Normalization Group |
| *O*_b_*X*_b_*Y*_b_*Z*_b_ | It represents absolute coordinate system |
| *O*_e_*X*_e_*Y*_e_*Z*_e_ | It represents relative coordinate system |
| *X*_b_, *Y*_b_, *Z*_b_ | *X*, *Y*, *Z* directions in the absolute coordinate system |
| *X*_e_, *Y*_e_, *Z*_e_ | *X*, *Y*, *Z* directions in the relative coordinate system |
| **Symbols** | |
| *V* | Control volume of the computational domain |
| *S* | Control surface of of the computational domain |
| $\mathop{W}\limits^{\to}$ | Conservation vector of the equation RANS |
| $\mathop{F}\limits^{\to}$ | Inviscid fluxes of the equation RANS |
| $\mathop{G}\limits^{\to}$ | Viscous fluxes of the equation RANS |
| $\mathop{Q}\limits^{\to}$ | Source fluxs of the equation RANS due to the source term |
| *t* | Current calculation time |
| *H* | Total enthalpies |
| *E_r_* | Total internal energy |
| *τ_xx_*, *τ_yy_*, *τ_zz_*, *τ_xy_*, *τ_xz_*, *τ_yz_*; $\Phi_{x}$, $\Phi_{y}$, $\Phi_{z}$ | Viscous quantities and heat fluxes in three directions respectively |
| $\mathop{q}\limits^{\to} _{w}$ | Grid velocity perpendicular to the mesh unit surface |
| $\mathop{q}\limits^{\to}$ | Airflow velocity vector |
| $u$,$\nu$,$\omega$ | Airflow velocity components in three directions |
| *ρ* | Airflow density |
| *p* | Airflow pressure |
| $\Omega$ | Rotational speed of the rotor |
| $u_{i}$ | Time mean velocity in the direction *i* |
| $x_{i}$ | Coordinate in the *i* direction |
| *k* | Turbulent kinetic energy |
| *ε* | Turbulent dissipation rate |
| $\mu_{eff}$ | Total viscosity of airflow |
| $\mu_{t}$ | Turbulent viscosity of airflow |
| $\mu$ | Kinematic viscosity of airflow |
| $G_{k}$ | The production term of turbulent kinetic energy *k* due to the average velocity gradient |
| $G_{b}$ | The production term of turbulent kinetic energy *k* caused by buoyancy |
| $C_{1\varepsilon}^{*}$ | An incremental modification of the RNG *k*-*ε* equation based on the Standard *k*-*ε* equation |
| $\eta$ | The ratio of turbulence to mean flow in time scale |
| $E_{ij}$ | Characteristic strain rate |
| $\alpha_{k}$,$\alpha_{\varepsilon}$ | Turbulence model constants |
| $C_{\mu}$,$C_{1\varepsilon}$,$\eta_{0}$,$\beta$,$C_{2\varepsilon}$ | Constant terms |

**3. What grid generation software is used? If the code is in-house, specific schemes should be explained, or published papers should be cited.**

**The authors' reply：**Thank you very much for the professional and detailed review comment from the reviewer. Dear reviewer, the whole grid partitioning process was described in detail in Section 2.3.3 of the revised version.

The meshes for all the computing domains were constructed in ANSYS Fluent Meshing (ANSYS 2023R1, Inc., USA), and the mesh models of Computing Domains 1, 2, 3 and 4 were constructed using the Poly-Hexcore **[26]** mesh strategy. To improve the calculation accuracy of the flight-coupled airflow, the minimum and maximum side length sizes were finally set at 0.003 m and 0.05m respectively, the growth rate of grid side length was set at 1.05, the curvature normal angle of complex surface was set at 10 degrees, the number of grid cells at small gaps was set to 3, and the grid number of the entire computing area eventually reached 4.598 million. The results showed that the maximum orthogonal quality parameters of surface mesh and volume mesh were 0.49 and 0.63 (much less than 1) respectively, which met the grid quality requirements. The surface grid distribution law for the four-rotor UAV is shown in Fig 6. To show the rotor surface grid more clearly, the grid distribution near the area was enlarged. All the modified parts in the revised version were marked in red.

**[26] Vo Duy-Tan, Mai Thanh Dam, Kim Byungwook, Ryu Jaiyoung. Numerical study on the influence of coolant temperature, pressure, and thermal barrier coating thickness on heat transfer in high-pressure blades. International Journal of Heat and Mass Transfer. 2022, 189, 122715.**

**Fig 6. Grid structure diagram for the solid** **surfaces of the four-rotor UAV.**

**4. What software is being used for the NS solver as well? In the case of in-house code, specific calculation methods (Space discretization methods (inviscid, viscous), time integration methods, pressure coupling, matrix solvers, etc.) should be explained or published papers should be cited. The reviewer speculates that some commercial software(StarCCM+ or ANSYS) is used.**

**The authors' reply：**Thank you very much for the professional and detailed review comment from the reviewer. As the reviewer pointed out, the CFD simulations were carried out by the ANSYS Fluent Double-Precision Solver(ANSYS 2023R1, Inc., USA) using RYZEN with AMD 9554 (64 cores, two pieces; 3.75 GHz; 768 GB RAM). A pressure-based solver was used to solve the plant protection UAV flight-coupled wind field, and the Coupled algorithm was used for unsteady simulations to calculate pressure-velocity coupling. Pressure was discretised with a second order scheme. Density, Momentum and Energy were discretised with a second order upwind scheme. turbulence quantities were discretised with a first order upwind scheme. The transient term was discretised with a second order implicit scheme. The convergence criteria was that the residuals of all variables were set as 1.0×10^-4^. All the modified parts in the revised version were marked in red at Section 2.4.

**5. I don't know which are the calculation results and which are the experimental results. Are they really compared?**

**The authors' reply：**Thank you very much for the professional and detailed review comment from the reviewer. As pointed out by the reviewer, feasibility verification of theoretical calculation was a prerequisite before detailed analysis.

The flight-coupled wind field testing site was shown in Fig 7a. The Bluetooth wireless wind velocity sensor (UNI-T) was applied to record the *Z*_b_-axis wind speed values of testing points (as shown in Figs 7a and 7d). The test points were set right under the rotor at the UAV flight path, the height of the test points from the rotor was 2.25 m, as shown in Fig 7d.

The flight direction of the four-rotor UAV was defined as the *X*_b_-axis negative direction, the direction straight down was defined as the *Z*_b_-axis negative direction, as shown in Fig 7e. The observation points (lines) for dynamic calculation of flight-coupled wind field were also illustrated in Fig 7e. In this paper, the dynamic theoretical calculations of the flight wind field under two working conditions were carried out. The first working condition was that the UAV hovered for 1.3 s, and then flew in the *X*_b_-axis negative direction at a speed of 3 m/s. The second working condition was that the UAV flew directly along the *X*_b_-axis negative direction at a speed of 3 m/s. Based on Working Condition 1, the flow field evolution law during the transition from hover to flight state was mainly studied; the wind field distribution law after flight stabilization was analyzed in Working Condition 2. According to the data provided by XAIRCRAFT, the flight speed of the four-rotor UAV was defined as 3 m/s, and the forward tilt angle of the UAV body was set as 9°. The running parameters of each rotor in Working Conditions 1 and 2 were shown in Tables 1 and 2 respectively. (This description was added and marked in red at Section 2.3.2(1) in the revised version.)

In addition, to better illustrate the relative error between experimental test (Testing Point) and theoretical calculation (Observation Point), Table 4 had been added to section 3.1 in the revised version. In Working Condition 1, the UAV hovered for 1.3s, and the distance of UAV flight was relatively short, so the ground effect had a great influence on the Zb-axis wind speed value of the Observation Points 1 and 2 (As shown in Fig 10d and 13b). In addition, the sampling frequency of the UNI-T wind speed sensor is relatively low (1 Hz). Therefore, only the Zb-axis velocity peaks of the testing point in wind field test and observation point in Working Condition 2 could be used for comparison. When the flight-coupled wind field swept over the Observation Point 1, the wind field was not completely stable. So the relative error of the wind speed peak in the *Z*_b_-axis for the testing point and Observation Point 2 (in Working Condition 2) was 12.7%. (This description was added and marked in red at Section 3.1 in the revised version.)

All the modified parts in the revised version were marked in red.

(b)

(c)

(a)

**(d)**

**(e)**

**Fig 7.** **Wind field test and calculation observation position setting.** …...(d) Layout of testing points of flight-coupled wind field (spray); (e) Layout diagram of observation lines (points) for dynamic calculation of flight-coupled wind field.

**Table 1. Running parameters of each rotor in Working Condition 1**

| Rotor number | Coordinates of the rotation center  in Working Condition 1 | | | | Angular velocity (rad/s) |
| --- | --- | --- | --- | --- | --- |
|  | *x /* m | | *y /* m | *z /* m |  |
|  | *t*≤1.3(s)（UAV Fixed） | *t*＞1.3(s)（UAV Flight） |  |  |  |
| 1 | -0.415239839 | -0.415239839-(3*t*-3.9) | -0.518860346 | -0.099685111 | 249.9 |
| 2 | -0.415239839 | -0.415239839-(3*t*- 3.9) | 0.518860347 | -0.099685111 | -249.9 |
| 3 | 0.541537512 | 0.541537512-(3*t*- 3.9) | 0.516302113 | 0.051853534 | 253 |
| 4 | 0.541537512 | 0.541537512-(3*t*- 3.9) | -0.516302112 | 0.051853534 | -253 |

**Table 2. Running parameters of each rotor in Working Condition** 2

| Rotor number | Coordinates of the rotation center  in Working Condition 2 | | | Angular velocity (rad/s) |
| --- | --- | --- | --- | --- |
|  | *x /* m （UAV Flight） | *y /* m | *z /* m |  |
| 1 | -0.415239839-3*t* | -0.518860346 | -0.099685111 | 249.9 |
| 2 | -0.415239839-3*t* | 0.518860347 | -0.099685111 | -249.9 |
| 3 | 0.541537512-3*t* | 0.516302113 | 0.051853534 | 253 |
| 4 | 0.541537512-3*t* | -0.516302112 | 0.051853534 | -253 |

**Table 4. Comparison of peak vertical velocity values between test points and observation points**

| Object | | Coordinates | | Peak vertical  velocity | | Coordinates | | | Peak vertical velocity | Absolute value of peak velocity relative error |
| --- | --- | --- | --- | --- | --- | --- | --- | --- | --- | --- |
|  |  | *y /* m | *z /* m | *V*_Z_-Peak */* (m/s) | Average value  */* (m/s) | *x /* m | *y /* m | *z /* m | *V*_Z_-Peak  */* (m/s) | Relative error  */* % |
| Wind field test | Testing Point 1 | -0.516302 | -2.25 | -8.0 | -7.9 | \ | \ | \ | \ | \ |
|  | Testing Point 2 | 0.516302 | -2.25 | -7.7 |  | \ | \ | \ | \ | \ |
| Calculation Working Condition 1 | Observation Point 1 | \ | \ | \ | \ | -2.536297 | -0.516302 | -2.25 | -7.5 | 5.1 |
|  | Observation Point 2 | \ | \ | \ | \ | -4.536297 | -0.516302 | -2.25 | -8.7 | 10.1 |
| Wind field test | Testing Point 1 | -0.516302 | -2.25 | -8.0 | -7.9 | \ | \ | \ | \ | \ |
|  | Testing Point 2 | 0.516302 | -2.25 | -7.7 |  | \ | \ | \ | \ | \ |
| Calculation Working Condition 2 | Observation Point 1 | \ | \ | \ | \ | -2.536297 | -0.516302 | -2.25 | -8.4 | 6.3 |
|  | Observation Point 2 | \ | \ | \ | \ | -4.536297 | -0.516302 | -2.25 | -8.9 | 12.7 |

(d)

**Fig 10. Horizontal and vertical section velocity distribution diagram at typical flight moments of the overall plant protection UAV (Working Condition 1).** …… (d) After 0.696 s of flight.

(b)

**Fig 13. Horizontal and vertical section velocity distribution diagram at typical flight moments after flight (No hover process) for the overall four-rotor UAV (Working Condition 2).** ……(b) After 1.742 s of flight.

**6. The boundary condition name, such as pressure outlet, does not tell what the boundary condition is(the software's proprietary words).**

**The authors' reply：**Thank you very much for the professional and detailed review comment from the reviewer. As pointed out by the reviewer, the boundary mathematical descriptions were not indicated in the boundary conditions of the software platform (ANSYS Fluent 2023R1, Inc., USA). In view of this, the setting of boundary conditions were explained in detail in Section 2.3.2(3) of the revised version.

The background operating pressure of the overall CFD model was 101325 Pa. The initial condition (*t* = 0 s) of the overall CFD model was that gauge pressure *p* = 0 Pa, temperature *T* = 300 K, three velocity components $u=v=w=0$. The boundary conditions (*t*≥0 s) for the outermost surfaces of the CFD model (at Computing Domains 3 and 4) and the solid surfaces inside the CFD model (at Computing Domains 1 and 2) in Figure 5 were set as shown in the following Table 3 and Fig 5.

**Table 3. Setting of boundary conditions**

| **Boundary surface name** | **Boundary type in ANSYS Fluent** | **Boundary type expressed in**  **mathematical expressions** |
| --- | --- | --- |
| Front surface in Domain 3 | Pressure Outlet | *p* = 0 |
| Front surface in Domain 4 |  |  |
| Back surface in Domain 3 |  |  |
| Back surface in Domain 4 |  |  |
| Left surface in Domain 3 |  |  |
| Left surface in Domain 4 |  |  |
| Right surface in Domain 3 |  |  |
| Right surface in Domain 4 |  |  |
| Top surface |  |  |
| Bottom surface (ground) | Wall (No Slip) | $u$=$\nu$=$\omega$=0, $\frac{\partial p}{\partial x}=\frac{\partial p}{\partial y}=\frac{\partial p}{\partial z}=0$  |
| UAV fuselage surfaces |  |  |
| UAV rotor surfaces |  |  |

**Fig 5. Schematic diagram of boundary condition setting.**

**7. It is necessary to explain what kind of boundary conditions are imposed on the pressure and velocity by using specific mathematical expressions.**

**The authors' reply：**Thank you very much for the professional and detailed review comment from the reviewer. As pointed out by the reviewer, the boundary mathematical descriptions were not indicated in the boundary conditions of the software platform (ANSYS Fluent 2023R1, Inc., USA). In view of this, the setting of boundary conditions were explained in detail in Section 2.3.2(3) of the revised version.

The background operating pressure of the overall CFD model was 101325 Pa. The initial condition (*t* = 0 s) of the overall CFD model was that gauge pressure *p* = 0 Pa, temperature *T* = 300 K, three velocity components $u=v=w=0$. The boundary conditions (*t*≥0 s) for the outermost surfaces of the CFD model (at Computing Domains 3 and 4) and the solid surfaces inside the CFD model (at Computing Domains 1 and 2) in Figure 5 were set as shown in the following Table 3 and Fig 5.

**Table 3. Setting of boundary conditions**

| **Boundary surface name** | **Boundary type in ANSYS Fluent** | **Boundary type expressed in**  **mathematical expressions** |
| --- | --- | --- |
| Front surface in Domain 3 | Pressure Outlet | *p* = 0 |
| Front surface in Domain 4 |  |  |
| Back surface in Domain 3 |  |  |
| Back surface in Domain 4 |  |  |
| Left surface in Domain 3 |  |  |
| Left surface in Domain 4 |  |  |
| Right surface in Domain 3 |  |  |
| Right surface in Domain 4 |  |  |
| Top surface |  |  |
| Bottom surface (ground) | Wall (No Slip) | $u$=$\nu$=$\omega$=0, $\frac{\partial p}{\partial x}=\frac{\partial p}{\partial y}=\frac{\partial p}{\partial z}=0$  |
| UAV fuselage surfaces |  |  |
| UAV rotor surfaces |  |  |

**Fig 5. Schematic diagram of boundary condition setting.**
