# Supplementary material for: The spatiotemporal evolution of flight-coupled wind field for a four-rotor plant protection unmanned aerial vehicle
Source: PLoS One. 2025 May 8;20(5):e0318769. doi: 10.1371/journal.pone.0318769 (PMC12061431; doi:10.1371/journal.pone.0318769)
Supplement: Attachment — Submitted filename: Response_to_Reviewers_auresp_2.docx [file pone.0318769.s004.docx]

**Reviewer #1:**

**1. The text needs to be checked for grammatical errors to improve on readers understanding of the information inside the paper. There are still some sentences that require improvement.**

**The authors' reply: Thank you very much for the professional and detailed review comment from the reviewer. As the reviewer pointed out, there are still some grammatical problems and unintelligible sentences. After this paper was read through several times, the unintelligible sentences have either been modified or expanded. For example, several sentences vary as follows:**

**In the abstract, “Only when the coupled wind field is analyzed can the interaction mechanism** [**among**](https://www.baidu.com/s?rsv_idx=1&wd=among&fenlei=256&usm=1&ie=utf-8&rsv_pq=ebe87f0c0042c620&oq=%E4%B8%89%E8%80%85%E4%B9%8B%E9%97%B4%E7%94%A8%E4%BB%80%E4%B9%88%E4%BB%8B%E8%AF%8D&rsv_t=3d55W7EhGDnfnuFvj0MUOU37GwYQkRRB0YNrztXQ1wSXkw940A6zfhv%2BsZw&sa=re_dqa_dda&icon=1) **wind field, pesticide droplet, and crop canopy be effectively studied, then the flight spray scheme can be reasonably formulated.” has been modified to “When the multi-rotor plant protection UAV flew and sprayed, the liquid droplets were accelerated, the stems and branches were shook, and the leaves were turned over in the disturbance area of spiral backward flight-coupled wind field. The combination of flight-coupled wind field, liquid droplets and crop canopy were closely related. Only when the flight-coupled wind field was analyzed could the interaction mechanism** [**among**](https://www.baidu.com/s?rsv_idx=1&wd=among&fenlei=256&usm=1&ie=utf-8&rsv_pq=ebe87f0c0042c620&oq=%E4%B8%89%E8%80%85%E4%B9%8B%E9%97%B4%E7%94%A8%E4%BB%80%E4%B9%88%E4%BB%8B%E8%AF%8D&rsv_t=3d55W7EhGDnfnuFvj0MUOU37GwYQkRRB0YNrztXQ1wSXkw940A6zfhv%2BsZw&sa=re_dqa_dda&icon=1) **flight-coupled wind field, liquid droplets, and crop canopy be effectively studied, then the flight and spray scheme could be reasonably formulated”.**

**In the abstract, “By combining Re-Normalization Group (RNG) k-ε turbulence model, compressible Reynolds Averaged Navier-Stokes (RANS) equations, dynamic mesh based on the spring smoothing and layering method, pressure-velocity coupling algorithm, this paper established a computational fluid dynamics (CFD) model of the flight-coupled wind field for the four-rotor plant protection UAV and discussed the dynamic evolution law of the flight-coupled wind field in the spatial and temporal dimensions.” has been modified to “By combining Re-Normalization Group (RNG) *k*-*ε* turbulence model, compressible Reynolds Averaged Navier-Stokes (RANS) equation, dynamic mesh based on the spring smoothing and layering method, pressure-velocity coupling algorithm, a computational fluid dynamics (CFD) model of the flight-coupled wind field for the four-rotor plant protection UAV was established, the dynamic evolution law of the flight-coupled wind field in the spatial and temporal dimensions was also discussed in this paper.”**

**In section 2.3.2 (1), “Adjusting the rotation speed of the four rotors can realize the adjustment of flight speed and flight attitude.” has been changed to “The adjustment of flight speed and flight attitude can be made by changing the rotation speed of the four rotors”.**

**In section 2.3.2 (2), “In general, through the setting of the above connection method, the numerical calculation of the dynamic flow field of the four-rotor plant protection UAV moving along the negative direction of the *X*_b_-axis at a speed of 3 m/s could be realized.” has been changed to “In general, through the setting of the above connection method,** **the dynamic numerical calculation of the flight-coupled wind field for the four-rotor plant protection UAV could be realized.”**

**In addition, as the whole paper was read through, some minor grammatical errors have also been corrected incidentally, such as the different descriptions of proper nouns and variables have been unified, the errors in the singular and plural expressions of nouns and verbs have been corrected, and the errors in the past tense and past participle of verbs were also corrected, et al. All changes in the revised version were marked in red.**

**2. Please check again the governing equations and their corresponding explanations. For example. F in the navier-stokes equation may not actual represent inviscid fluxes.**

**The authors' reply: Thank you very much for the professional and detailed review comment from the reviewer. As the reviewer pointed out, the corresponding explanations of the governing equation in the previous version was not particularly reasonable. In view of this, after consulting many relevant professional literature, the corresponding explanations of every term for the governing equation had been revised as follows (All the modified explanations in the revised version were marked in red. The corresponding parameters in the Nomenclature Table had also been modified):**

 **(1)**

$\mathop{W}\limits^{\to} =\left[\begin{matrix} \rho\\ \rho u \\ \rho\nu\\ \rho\omega\\ \rho E_{r} \end{matrix}\right]$, $\mathop{F}\limits^{\to} =\left[\begin{matrix} \rho(\mathop{q}\limits^{\to} -\mathop{q}\limits^{\to} _{w}) \\ \rho u(\mathop{q}\limits^{\to} -\mathop{q}\limits^{\to} _{w})+p\mathop{i_{x}}\limits^{\to} \\ \rho\nu(\mathop{q}\limits^{\to} -\mathop{q}\limits^{\to} _{w})+p\mathop{i_{y}}\limits^{\to} \\ \rho\omega(\mathop{q}\limits^{\to} -\mathop{q}\limits^{\to} _{w})+p\mathop{i_{z}}\limits^{\to} \\ \rho H(\mathop{q}\limits^{\to} -\mathop{q}\limits^{\to} _{w}) \end{matrix}\right]$,, $\mathop{Q}\limits^{\to} =\left[\begin{matrix} 0 \\ \text{-}\rho\omega\Omega\\ 0 \\ \rho u\Omega\\ 0 \end{matrix}\right]$ **(2)**

**where *V* is the control volume of discrete unit in the computing domain; *S* is the control surface of discrete unit in the computing domain;** $\mathop{W}\limits^{\to}$ **is the conserved variable of the RANS equation; the** $\mathop{F}\limits^{\to}$ **is the convective flux of the RANS equation;** $\mathop{G}\limits^{\to}$ **is the diffusion flux of the RANS equation;** $\mathop{Q}\limits^{\to}$ **is the source flux due to the rotation of the rotors.**

**3. Please check again that all the abbreviations and symbols in the text are defined. For example, *y*+.**

**The authors' reply：Thank you very much for the professional and detailed review comment from the reviewer. In order to increase the readability of the paper, a “Nomenclature” table was added at the beginning of the revised version, as shown below. According to the opinion of the reviewer, some variables were not defined in the text or were not recorded in the table below. After checking the full paper again, it was found that *y*+ was not defined in the paper. And *y*+, △*y,*** $\tau_{w}$ **were not recorded in the table below. Therefore, these mistakes have been corrected in the modified version and marked in red.**

 **(8)**

**where *y*+ is a dimensionless number that characterizes whether the first-layer grid is reasonable;** $\Delta y$ **is the thickness of the first boundary layer;** $\rho$ **and** $\mu$ **are the density and viscosity of the fluid within the first boundary layer mesh;** $\tau_{w}$ **is the shear stress on the solid wall of the first layer.**

| **Nomenclature** | |
| --- | --- |
| **Abbreviations** | |
| UAV | Unmanned Aerial Vehicle |
| CFD | Computational Fluid Dynamics |
| RANS | Reynolds Averaged Naviere-Stokes |
| RNG | Re-Normalization Group |
| *O*_b_*X*_b_*Y*_b_*Z*_b_ | It represents absolute coordinate system |
| *O*_e_*X*_e_*Y*_e_*Z*_e_ | It represents relative coordinate system |
| *X*_b_, *Y*_b_, *Z*_b_ | *X*, *Y*, *Z* directions in the absolute coordinate system |
| *X*_e_, *Y*_e_, *Z*_e_ | *X*, *Y*, *Z* directions in the relative coordinate system |
| **Symbols** | |
| *V* | Control volume of discrete unit in the computing domain |
| *S* | Control surface of discrete unit in the computing domain |
| $\mathop{W}\limits^{\to}$ | Conservation variables of the RANS equation |
| $\mathop{F}\limits^{\to}$ | Convective fluxs of the RANS equation |
| $\mathop{G}\limits^{\to}$ | Diffusion fluxes of the RANS equation |
| $\mathop{Q}\limits^{\to}$ | Source fluxs due to the rotation of the rotors |
| *t* | Current calculation time |
| *H* | Total enthalpies |
| *E_r_* | Total internal energy |
| *τ_xx_*, *τ_yy_*, *τ_zz_*, *τ_xy_*, *τ_xz_*, *τ_yz_*; $\Phi_{x}$, $\Phi_{y}$, $\Phi_{z}$ | Viscous quantities and heat fluxes in three directions respectively |
| $\mathop{q}\limits^{\to} _{w}$ | Grid velocity perpendicular to the mesh unit surface |
| $\mathop{q}\limits^{\to}$ | Airflow velocity vector |
| $u$,$\nu$,$\omega$ | Airflow velocity components in three directions (*x, y, z*) |
| *ρ* | Airflow density |
| *p* | Airflow pressure |
| $\Omega$ | Rotational speed of the rotor |
| $u_{i}$ | Time mean velocity in the direction *i* |
| $x_{i}$ | Coordinate in the *i* direction |
| *k* | Turbulent kinetic energy |
| *ε* | Turbulent dissipation rate |
| $\mu_{eff}$ | Total viscosity of airflow |
| $\mu_{t}$ | Turbulent viscosity of airflow |
| $\mu$ | Kinematic viscosity of airflow |
| $G_{k}$ | The production term of turbulent kinetic energy *k* due to the average velocity gradient |
| $G_{b}$ | The production term of turbulent kinetic energy *k* caused by buoyancy |
| $C_{1\varepsilon}^{*}$ | An incremental modification of the RNG *k*-*ε* equation based on the Standard *k*-*ε* equation |
| $\eta$ | The ratio of turbulence to mean flow in time scale |
| $E_{ij}$ | Characteristic strain rate |
| $\alpha_{k}$,$\alpha_{\varepsilon}$ | Turbulence model constants |
| $C_{\mu}$,$C_{1\varepsilon}$,$\eta_{0}$,$\beta$,$C_{2\varepsilon}$ | Constant terms |
| *y*+ | A dimensionless number that characterizes whether the first-layer grid is reasonable |
| △*y* | The thickness of the first boundary layer |
| $\tau_{w}$ | The shear stress on the solid wall of the first layer |

**4. If possible, please use the standard terms usually used in explaining computational fluid dynamics simulation. For example, control body can be replaced by control** **volume.**

**The authors' reply: Thank you very much for the professional and detailed review comment from the reviewer. As the reviewer pointed out, the corresponding explanations of the governing equation in the previous version was not particularly reasonable. Dear reviewer, after consulting many relevant professional literature, the corresponding explanations of every term for the governing equation had been revised as follows.**

**In section 2.1, the “flight-coupled airflow area” was changed to “flight-coupled wind Field ”, “*V* is the control body of the computational domain” was changed to “*V* is the control volume of discrete unit in the computing domain”, “*S* is the control surface of the computational domain” was changed to “*S* is the control surface of discrete unit in the computing domain”, “**$\mathop{W}\limits^{\to}$**is the conservation vector of the equation” was changed to “**$\mathop{W}\limits^{\to}$ **is the conserved variable of the RANS equation”, “the**$\mathop{F}\limits^{\to}$**and**$\mathop{G}\limits^{\to}$**is the inviscid and viscous fluxes of the equation” was changed to “the** $\mathop{F}\limits^{\to}$ **is the convective flux of the RANS equation” and “**$\mathop{G}\limits^{\to}$ **is the diffusion flux of the RANS equation”, “**$\mathop{Q}\limits^{\to}$**is the source flux due to the source term” was changed to “**$\mathop{Q}\limits^{\to}$ **is the source flux due to the rotation of the rotors”. All the modified explanations in the revised version were marked in red (The corresponding parameters in the Nomenclature Table had also been modified).**

**5. Please make sure that the terms used in the text are always consistent. For example, turbulent kinetic energy *k* and turbulence *ε* equation; epsilon equation and dissipation equation.**

**The authors' reply: Thank you very much for the professional and detailed review comment from the reviewer. As the reviewer pointed out, some formula variables and nouns were expressed in more than one way in paper. In view of this, after reading through the whole paper, the following content had been revised as follows:**

**The description of the variable *k* and ε had all been changed to the “turbulent kinetic energy *k*” and “turbulent dissipation rate *ε*” in the “section 2.2” and “Nomenclature Table”. The “flight-coupled airflow” had all been changed to “flight-coupled wind field” throughout the paper. All changes in the revised version were marked in red.**

**6. It may be useful to make some indications/labels on the results/ figures itself corresponding to the explanation provided in the text. For example, in Figure 10, which is the UV body, airflow, horseshoe tail, etc.....which would really assist the readers in understanding the findings even more.**

**The authors' reply: Thank you very much for the professional and detailed review comment from the reviewer. According to the opinion of the reviewer, some indications/labels were added in the Fig 12, Fig 15, Fig 10, Fig 16 after the full paper was checked. In this way, the descriptions in the text and the labels in the figures could be well matched. All the modified figures in the revised version were marked in red.**

**“Vortex areas” at the tail of the UAV body**

**The UAV body**

**Fig 12. Horizontal streamline diagram under velocity background at 0.696 s (Working Condition 1).**

**α_1_=72°**

**α_2_=69°**

**α_3_=61°**

**α_4_=56°**

**Fig 15. The angles between the horizontal direction and the four curves formed by *Z*_b_-direction velocity peaks (Working Condition 2)**

(a)

(b)

(c)

(d)

**UAV body**

**Lateral interruption**

**Downwash airflow**

**Flight-coupled wind field**

**Four circular wind fields**

**Ground effect airflow**

**Initial “four horseshoe tails”**

**Developing “four horseshoe tails”**

**Dynamically stable**

**“four horseshoe tails”**

**Fig 10. Horizontal and vertical section velocity distribution diagram at typical flight moments of the overall plant protection UAV (Working Condition 1).** (a) After 1.3 s of hover; (b) After 0.245 s of flight; (c) After 0.446 s of flight; (d) After 0.696 s of flight.

**The diagonally downward "wind wall"**

**The forward flow areas**

**Fig 16. Longitudinal streamline diagram under velocity background at 2.492 s (Working Condition 2).**

**7. For Figure 6, please make the figure larger so that the readers may see the mesh/grid more clearly.**

**The authors' reply：Thank you very much for the professional and detailed review comment form the reviewer. In accordance with the opinion of the reviewer, Fig 6 had been enlarged so that the readers could see the mesh/grid more clearly.**

**Partial enlarged image**

**Rotors**

**UAV body**

**Centrifugal nozzle**

**Fig 6. Grid structure diagram for the solid surfaces of the four-rotor UAV.**

**Reviewer #2:**

**1. There are numbers in the paper with a very large number of significant digits, but it is hard to have such precision in either the experimental or calculated values. All numbers need to be revised to show each with reliable precision.**

**The authors' reply: Thank you very much for the professional and detailed review comment from the reviewer. As the reviewer pointed out, there are numbers in the Table 1, 2, 4 with a very large number of significant digits. Dear reviewer, it could be seen from the table 1, 2, 4 that the physical quantities “Angular velocity”, “Peak velocity (*V*_Z_-Peak, Average value)”, “Relative error” had no more than 2 significant digits after the decimal point. While the physical quantities “Coordinates of the rotation center (*x, y, z*)”**, **“Coordinates (test points and observation points)” had more than 6 significant digits after the decimal point. This is because the plant protection UAV had a certain inclination angle of 9° when flying forward with a speed of 3m/s, the position coordinates between each rotor of the plant protection UAV would change, and the significant digits after the decimal point of the position coordinates for each feature point were the position recognition accuracy of the ANSYS Fluent, and the highest accuracy of the position coordinates reached 10^-9^m.**

**Table 1. Running parameters of each rotor in Working Condition** 1

| Rotor number | **Coordinates of the rotation center**  in Working Condition 1 | | | | **Angular velocity (rad/s)** |
| --- | --- | --- | --- | --- | --- |
|  | ***x /* m** | | ***y /* m** | ***z /* m** |  |
|  | *t*≤1.3(s)（UAV Fixed） | *t*＞1.3(s)（UAV Flight） |  |  |  |
| 1 | -0.415239839 | -0.415239839-(3*t*-3.9) | -0.518860346 | -0.099685111 | 249.9 |
| 2 | -0.415239839 | -0.415239839-(3*t*- 3.9) | 0.518860347 | -0.099685111 | -249.9 |
| 3 | 0.541537512 | 0.541537512-(3*t*- 3.9) | 0.516302113 | 0.051853534 | 253 |
| 4 | 0.541537512 | 0.541537512-(3*t*- 3.9) | -0.516302112 | 0.051853534 | -253 |

**Table 2. Running parameters of each rotor in Working Condition** 2

| Rotor number | Coordinates of the rotation center  in Working Condition 2 | | | **Angular velocity (rad/s)** |
| --- | --- | --- | --- | --- |
|  | ***x /* m** （UAV Flight） | ***y / m*** | ***z / m*** |  |
| 1 | -0.415239839-3*t* | -0.518860346 | -0.099685111 | 249.9 |
| 2 | -0.415239839-3*t* | 0.518860347 | -0.099685111 | -249.9 |
| 3 | 0.541537512-3*t* | 0.516302113 | 0.051853534 | 253 |
| 4 | 0.541537512-3*t* | -0.516302112 | 0.051853534 | -253 |

**Table 4. Comparison of peak vertical velocity values between test points and observation points**

| Object | | Coordinates | | Peak vertical  velocity | | Coordinates | | | Peak vertical velocity | Absolute value of peak velocity relative error |
| --- | --- | --- | --- | --- | --- | --- | --- | --- | --- | --- |
|  |  | ***y /* m** | ***z /* m** | ***V*_Z_-Peak */* (m/s)** | **Average value**  ***/* (m/s)** | *x /* m | *y /* m | *z /* m | ***V*_Z_-Peak**  ***/* (m/s)** | **Relative error**  ***/* %** |
| Wind field test | Testing Point 1 | -0.516302 | -2.25 | -8.0 | -7.9 | \ | \ | \ | \ | \ |
|  | Testing Point 2 | 0.516302 | -2.25 | -7.7 |  | \ | \ | \ | \ | \ |
| Calculation Working Condition 1 | Observation Point 1 | \ | \ | \ | \ | -2.536297 | -0.516302 | -2.25 | -7.5 | 5.1 |
|  | Observation Point 2 | \ | \ | \ | \ | -4.536297 | -0.516302 | -2.25 | -8.7 | 10.1 |
| Wind field test | Testing Point 1 | -0.516302 | -2.25 | -8.0 | -7.9 | \ | \ | \ | \ | \ |
|  | Testing Point 2 | 0.516302 | -2.25 | -7.7 |  | \ | \ | \ | \ | \ |
| Calculation Working Condition 2 | Observation Point 1 | \ | \ | \ | \ | -2.536297 | -0.516302 | -2.25 | -8.4 | 6.3 |
|  | Observation Point 2 | \ | \ | \ | \ | -4.536297 | -0.516302 | -2.25 | -8.9 | 12.7 |

**2. In Figure 8, the caption states that the calculation results are plotted, but for the other figures, it is not clear whether they draw** **measurement results or calculation results. Figure 9 says that it compares experimental and calculated values, but it is not clear which lines are experimental values and which are calculated values.**

**The authors' reply: Thank you very much for the professional and detailed review comment from the reviewer. As the reviewer pointed out, the dynamic evolution law of the flight-coupled wind field in the spatial and temporal dimensions were described in Figure 8, 9, 10, 11, 12, 13, 14, 15, 16, and in Table 4. Among them, the Figure 8, 10, 11, 12, 13, 14, 15, 16 were the theoretical calculation results, Figure 9 and Table 4 were the comparison of test data and theoretical calculation results. In Figure 8, the Figure 8(a) and Figure 8(b) were the *Z*_b_-axis velocity values at characteristic points of numerical calculation, the Figure 8(c) were the *Z*_b_-axis velocity values at characteristic points of wind speed measurement experiment. The illustrations of Figure 8, 9, 10, 11, 12, 13, 14, 15, 16 had all been modified and marked in red in the revised version**

**3. In several sentences, it is difficult to understand the meaning. For example, "Only when the coupled wind field is analyzed can the interaction mechanism among wind field, pesticide droplet, and crop canopy be effectively studied, then the flight spray scheme can be reasonably formulated." in the abstract.**

**The authors' reply：Thank you very much for the professional and detailed review comment from the reviewer. As the reviewer pointed out, the relationship between the flight-coupled wind field, liquid droplets, and crop canopy was not explained, and a summary sentence was given directly, which made the meaning of the sentence very stiff. As a result, the paragraph in the abstract had been modified as follow (the modifications were marked in red in the revised version):**

**“When the multi-rotor plant protection UAV flew and sprayed, the liquid droplets were accelerated, the stems and branches were shook, and the leaves were turned over in the disturbance area of spiral backward flight-coupled wind field. The combination of flight-coupled wind field, liquid droplets and crop canopy were closely related. Only when the flight-coupled wind field was analyzed could the interaction mechanism** [**among**](https://www.baidu.com/s?rsv_idx=1&wd=among&fenlei=256&usm=1&ie=utf-8&rsv_pq=ebe87f0c0042c620&oq=%E4%B8%89%E8%80%85%E4%B9%8B%E9%97%B4%E7%94%A8%E4%BB%80%E4%B9%88%E4%BB%8B%E8%AF%8D&rsv_t=3d55W7EhGDnfnuFvj0MUOU37GwYQkRRB0YNrztXQ1wSXkw940A6zfhv%2BsZw&sa=re_dqa_dda&icon=1) **flight-coupled wind field, liquid droplets, and crop canopy be effectively studied, then the flight and spray scheme could be reasonably formulated.”**
